# SLPDBO-BP: an efficient valuation model for data asset value

**DOI:** 10.7717/peerj-cs.2813

**Published:** 2025-04-30

**Authors:** Cuiping Zhou, Shaobo Li, Cankun Xie, Panliang Yuan, Zihao Liao

**Affiliations:** 1 Guizhou University, State Key Laboratory of Public Big Data, Guiyang, Guizhou, China; 2 Guizhou Institute of Technology, Guiyang, Guizhou, China

**Keywords:** Dung beetle optimizer, BP neural network, SLPDBO-BP, Data assets, Value assessment

## Abstract

Data asset value assessment is of strategic significance to the development of data factorization, in order to solve the problems of strong assessment subjectivity and low assessment efficiency and accuracy in traditional assessment methods. This article introduces the SLPDBO-BP data asset assessment model for data asset value assessment. Firstly, the sinusoidal chaos mapping strategy, the Levy flight strategy and the fusion of adaptive weight variation operators are integrated to increase the population diversity of the algorithm, broaden the search range, and augment the global optimization capability of the algorithm. Secondly, in an attempt to comprehensively evaluate the optimization performance of SLPDBO, a series of numerical optimization experiments are carried out with 20 test functions and with popular optimization algorithms and dung beetle optimizer (DBO) algorithms with different improvement strategies. Finally, in order to verify the effectiveness of the proposed algorithm in data asset value assessment, the SLPDBO algorithm is combined with backpropagation (BP) to establish the SLPDBO-BP model for data asset value assessment, and the acquired data sets are used in the proposed model for data asset value assessment. The experimental results show that the SLPDBO-BP model performs well in assessment accuracy, and its assessment indexes mean absolute error (MAE), root mean square error (RMSE) and mean absolute percentage error (MAPE) are reduced by 35.1%, 37.6% and 38.7%, respectively, compared with the dung beetle optimizer backpropagation (DBO-BP) model, and its evaluation efficiency is improved, and the proposed model demonstrates better evaluation simulation effects by remarkably outperforming other models in terms of evaluation accuracy and error level.

## Introduction

Nowadays, with the rapid development of the technology-driven digital integrated economic environment, data elements play an increasingly important role in it. Data value allocation is an effective form of reflecting data factorization, while data value assessment is a key driver of data factor value release (Bendechache et al., 2023). A data asset is a data resource formed after the processing of user behavior information, public information, and other relevant information obtained through legal means with the help of modern computer technology. The value of data asset is the economic benefits or potential benefits brought to enterprises or individuals by making data an asset in a specific way. Its core lies in the fact that data can create value in various ways. The ability and level of enterprise data asset management will be linked to the degree of data utilization and the degree of data value release (Liang et al., 2024). Meanwhile, more and more enterprises are incorporating data asset management into their strategic planning. Data asset valuation has not only an economic role but also a prominent strategic role in modern industry. It can not only help enterprises optimize resource allocation, enhance investment grey, promote data trading and sharing, but also enhance their competitive advantages, develop and innovate products and services, and formulate effective data strategy plans to eventually realize the digital transformation of enterprises. Data value assessment can not only achieve data resourcing, data assessment, to data productization circulation empowerment (Fleckenstein, Obaidi & Tryfona, 2023), it can also provide management with powerful decision support to help them better understand the potential value and risk of data (Chang, Huang & Wu, 2024). Meanwhile, it can also provide enterprises with a more effective allocation of resources to improve market competitiveness while maximizing their economic benefits (Liu & Qiao, 2021), stimulate innovativeness, and motivate enterprises to develop new products and services, and ensure that companies can invest in high-value data assets.

Generally speaking, the methods used to evaluate data assets are divided into three main categories, including traditional methods, economics methods, and comprehensive methods. Among the traditional methods, the cost method (Fattinnanzi et al., 2020), the market method (Yeh, 2024), and the income method (Acuna et al., 2020) are representative. The classic economics assessment methods include game theory method (Gomes, Gutierrez & Ribeiro, 2023), real options method (Khodayari & Ranjbar, 2019). The main representatives of comprehensive methods include expert scoring method (Guo et al., 2024), hierarchical analysis method (Lim et al., 2024), fuzzy comprehensive evaluation method (Li, 2020) and so on. As digital technology continues to develop, the popularity of e-commerce and social media has led to a significant increase in the volume of data. Traditional data asset value assessment methods are often susceptible to ignoring certain areas of demand, lack full exploration of potential value, and are limited by manpower and existing empirical knowledge, and have a certain degree of subjectivity. Therefore, machine learning-based data asset value assessment methods are gradually gaining popularity among researchers. The method of machine learning is a modern assessment means of value analysis and prediction of data assets using machine learning technology data asset value assessment, mainly including neural networks (Yang et al., 2023), machine learning (Deppner et al., 2023) and other assessment methods. With the increasing scale and complexity of data, these advanced technologies have the characteristics of automation and efficiency, show high dynamic adaptability and excellent pattern recognition ability, can automatically process a large amount of data, multi-dimensional analysis, thus significantly reducing manual intervention, reducing the subjectivity of the assessment, and can more accurately capture the potential value of the data, and provide powerful support for the enterprise in the data-driven decision-making.

The backpropagation (BP) neural network is a machine learning method that captures complex nonlinear relationships between inputs and outputs (Bi & Wang, 2024), and is widely used in a variety of fields due to its flexibility and powerful modelling capabilities. However, BP neural networks still face the challenge of easily trapping into local optimal solutions, which ultimately leads to a reduction in prediction accuracy, and thus it is particularly important to optimize for its weights and thresholds to enhance the overall performance and generalization ability of the model, and ultimately achieve an increase in assessment precision and accuracy. To address the optimal design problem, researchers have introduced meta-heuristic algorithms (MA) (Abualigah et al., 2022) to solve the problem, such as grey wolf optimization algorithm (GWO) (Mirjalili, Mirjalili & Lewis, 2014), rime optimization algorithm (RIME) (Su et al., 2023) and multi-verse optimizer (MVO) (Mirjalili, Mirjalili & Hatamlou, 2016), which are effective in improving the process of adjusting the weights. Through these optimization techniques, the global search ability of the model can be enhanced to help it jump out of the local optimal solution, thus achieving higher prediction accuracy and broadening its application in practical problems. With the continuous deepening of research, researchers have offered many algorithms to solve optimization tasks in recent years, such as PID-based search algorithm (PSA) (Gao, 2023), snow ablation optimizer (SAO) (Deng & Liu, 2023) and sinh cosh optimizer (SCHO) (Bai et al., 2023). These algorithms have been widely used in numerical optimization and engineering applications with excellent results. Based on the characteristics of MA’s easy operation and strong seeking ability, this study introduces MA to optimize the weights and thresholds of BP neural networks to improve the precision and accuracy of data asset value assessment.

In 2023, Xue & Shen (2023) inspired by the rolling, dancing, foraging, stealing and breeding behavior of dung beetles, proposed a novel population intelligence optimization algorithm, dung beetle optimizer (DBO) (Xue & Shen, 2023), to deal with numerical optimization and practical applications. Although the DBO has the characteristics of strong seeking ability and fast convergence speed, considering the shortcomings of the DBO algorithm that it has insufficient global searching ability and tends to converge to the local optimum, researchers have done corresponding improvement work on it and applied it to different fields. Qiao et al. (2024) introduced the original DBO improved by two strategies, Levy flight and variable helix, and predicted the infrared radiation characteristics of axisymmetric nozzles using the IDBO-HKELM model. Lyu, Jiang & Yang (2024) use a combination of four strategies to improve DBO performance and apply it to the UAV 3D path planning problem. Wu, Xu & Liu (2024) applied a comprehensive and improved IDBO algorithm for traffic identification data analysis and feature selection in network traffic identification. Baris, Yanarates & Altan (2024) used an African Condor optimization algorithm based on tent chaotic mapping to estimate parameters and construct a highly accurate wind speed prediction model to improve wind energy efficiency. Restrepo-Cuestas & Montano (2024) developed optimization techniques for the solar cell parameter problem in the Bishop model to help solve the parameter estimation problem in the PV model and improve the quality and efficiency of solving the parameter optimization of the PV model. Karasu & Altan (2022) proposed a new crude oil price forecasting model based on technical indicators such as trend, volatility and momentum using chaotic Henry gas solubility optimization technique to cope with the chaotic and nonlinear problems of crude oil time series. According to the No Free Lunch (NFL) theorem (Sharma et al., 2022), there is no single optimal algorithm that can solve all problems effectively and efficiently. Different algorithms have the ability to solve different types of optimization problems (Lynn & Suganthan, 2017). Therefore, to address the problem that the weights and thresholds of BP neural networks need to be further optimal, this article proposes a multi-strategy improved DBO algorithm to make up for the shortcomings of the traditional DBO algorithm, so as to enhance the prediction accuracy of the final data asset assessment model. The main contributions of this article are concluded as follows:

(1) Based on the original DBO, a multi-strategy DBO (SLPDBO) that incorporates sinusoidal chaos mapping, Levy flight and particle swarm optimization algorithm (PSO) (Liang et al., 2023) fusion of adaptive weights and variational operators is introduced as an optimal strategy.

(2) The superiority of SLPDBO algorithm is verified by 20 test functions, and the obtained results are compared with popular algorithms and algorithms with different improvement strategies, and statistical tests are performed to verify that SLPDBO exceeds other competitors’ algorithms in terms of solution accuracy and robustness.

(3) The evaluation results of data asset transaction data are used to verify the effectiveness of the SLPDBO-BP model suggested in this article in solving practical problems and the efficiency of evaluation.

This document is organized as follows: “Related Work” describes related work. “Technical Background” introduces the basic DBO algorithm in detail and a multi-strategy enhanced dung beetle optimizer (SLPDBO) is developed and the improvement strategies are described. In “Experimental Tests and Analysis of Results”, the evaluation of the optimal performance of SLPDBO on 20 benchmark test functions is presented. “Empirical Analysis of Data Asset Valuation” verifies the effectiveness of the SLPDBO-BP data asset value assessment model in practical applications and the efficiency of the assessment by evaluating the data from the Youe dataset network. In “Conclusion and Future Research”, the experimental results are concluded and discussed.

## Related work

In today’s fast-growing data economy, we are in an era of data explosion. With the popularity of the Internet, the Internet of Things, social media and smart devices, there is a constant flow of data of all kinds. Enterprises, governments and individuals are generating, collecting and storing huge amounts of data every day. Therefore, data valuation has become an important engine to promote the development of data economy, and data value assessment has become a hot spot of contemporary research. Past research has mainly focused on the traditional cost element, market element and revenue element, providing an important theoretical foundation and practical guidance for data asset value assessment. Fattinnanzi et al. (2020) successfully estimated the asset value of an ancient Roman public building using the depreciated replacement cost (DRC) method and explored its market value, demonstrating an innovative application in the valuation of historic buildings. Odolinski et al. (2023) investigated the short-run marginal cost (SRMC) pricing principle and verified the feasibility of the EU track access pricing legislation through an econometric approach to provide empirical support for policy development. Yeh (2024) explored the assessment of intrinsic value of stocks using residual income model (RIM) and growth value model (GVM) by determining the parameters through regression analysis and market data. Although these studies have made some progress in their respective fields, some limitations remain. Firstly, traditional methods often fail to adequately take into account the dynamic nature and complexity of data, which may lead to one-sided assessment results. Secondly, some of the models used may lack universality in specific contexts and are difficult to adapt to the rapidly changing market environment. In addition, the availability and accuracy of historical and market data may also affect the reliability of the valuation results.

In order to adapt to the ever-changing data environment, researchers are committed to establishing systematic theoretical frameworks and constructing various valuation models in order to comprehensively understand the characteristics of data assets, their sources of value and their applications in different scenarios. These frameworks usually integrate knowledge from multiple disciplines, such as economics, management, and information technology, and also have made a lot of achievements in data asset value assessment. Harish et al. (2021) provided an effective tool for companies to perform digital asset valuation and risk assessment through a logistics financing platform (Log-Flock), advancing the practical application in the field of logistics financing (Harish et al., 2021) but his study may be affected by the quality of the platform’s data and the adaptability of its users, which limits its broad applicability. Kim & Min (2023) used panel regression to analyze the long-term determinants of the valuation effect of fixed asset factors, which provided cross-country data support and enriched the theoretical basis of fixed asset valuation, but also suffered from the drawback of failing to fully take into account the dynamic changes of other economic variables. Li (2020) employed fuzzy theory and integrated valuation methods to assess the assets of coastal enterprises through EVA modelling, proving the applicability of his methodology and enhancing the flexibility and accuracy of traditional valuation techniques, but may still have limitations when dealing with high levels of uncertainty. Liu & Zhang (2022) constructed a carbon asset assessment model for power enterprise projects based on real options model using multimodal knowledge mapping and real options model. The digital assessment of corporate carbon assets has promoted the research progress of sustainable development and carbon management, but its complexity may lead to implementation difficulties in practical applications.

Kim & Lee (2023) successfully achieved efficient valuation of human activity recognition based on inertial measurement unit (IMU) data by introducing a feature extraction structure and a meta-reinforcement learning-based algorithm to improve the performance and accuracy of the assessment model and achieve excellent assessment performance, but its complexity may limit the generality in practical applications. Veldkamp (2023) explored tools for data measurement and evaluation and examined the private value of data, providing new perspectives on the understanding of data assets, but the utility and adaptability of his work still needs to be further validated. The data-driven automated valuation framework proposed by Wu et al. (2022) combines geographic information systems (GIS) and neural network techniques to efficiently value real estate assets, demonstrating the potential of technology convergence in asset valuation (Wu et al., 2022), but it may face the problem of data heterogeneity when dealing with different types of real estate assets. Birch, Cochrane & Ward (2021) analyses the process by which personal data is transformed into an asset and explores the practices of large tech companies in accounting for, managing and valuing personal data, advancing the understanding of the data economy, but may not be sufficiently comprehensive in its considerations to have applicability issues when dealing with other types of companies. Chen, Pelger & Zhu (2024) enriches the asset pricing theory by constructing an asset pricing model using no-arbitrage conditions and deep neural networks to extract the macroeconomic states and identify the key factors affecting individual stock returns, identifying the key factors driving asset prices. However, its accuracy and applicability may be limited in a dynamic market environment. In summary, in data asset value assessment, previous research is mainly based on traditional assessment methods, which are more subjective and less efficient and precise, or assess tangible assets and other types of wireless assets, with less involvement in the assessment of data assets, as well as a lack of efficient data asset assessment models. To solve these problems, this article proposes an SLPDBO-BP data asset value assessment model, which uses the multi-strategy improved DBO algorithm to optimize the weights and thresholds of the BP neural network in order to enhance the data asset value assessment.

## Technical background

### Equations dung beetle optimization algorithm

Dung beetle optimization (DBO) implements an optimization process patterned after the dung beetle’s navigational and food-sourcing mechanisms, proposed in 2022 developed through studying and replicating the coping mechanisms and adaptation strategies of dung beetles in the natural world (Wu, Luo & Zhou, 2024). It mainly consists of four parts: rolling behavior, breeding behavior, foraging behavior, and stealing behavior (Wang et al., 2024). Its different subpopulations execute different search methods, enabling efficient discovery and space exposure. The specific behavioral process is as follows:

(1) Rolling behavior

Accessibility: Dung beetles use the sun as a guide for maintaining the roll of a ball of dung within a vertical line, where the strength of illumination affects the route taken by dung beetles, and the location of the rolled ball will be updated in the following way:

(1) $$\left\{ {\matrix{ {{x_r}({t_p} + 1) = {x_r}({t_p}) + a \cdot m \cdot {x_r}({t_p} - 1) + c \cdot {\rm \Delta}{x_r}} \hfill \cr {{\rm \Delta}{x_r} = \left| {{x_r}(t) - {X_g}^{\rm W}} \right|} \hfill \cr } } \right.$$

where: ${t_p}$ is the number of iterations, ${x_r}({t_p})$ is the position information of the first dung beetle in the first iteration, $m$ is the perturbation coefficient, $m \in \left( {0, 2} \right]$, which is taken as 0.3 in the code, $c$ is the fixed value in [0,1], which is taken as 0.1 in the code; $a$ denotes an indication of either the dung beetle departed his original roll direction or not, $a$ which with the likelihood value equal to 1 or −1, in which 1 means non-divergence and −1 means divergence; ${X_g}^{\rm W}$ is the poorest placement among the global rolling direction; ${\rm \Delta}{x_r}$ is the amount of change in light intensity, whose more considerable value indicates that it is farther away from the light source and the weaker the light source is.

Obstacle situation: When dung beetle meets an obstacle in the way, they will get a new rolling orientation with the behavior of dancing. When it encounters an obstacle, the tangent function is used to obtain a different scrolling direction to emphasize the appearance of the dung beetle’s dancing behavior. The rolling dung beetle position is updated in the following way:

(2) $${x_r}({t_p} + 1) = {x_r}({t_p}) + \tan \beta |{x_r}({t_p}) - {x_r}({t_p} - 1)|$$

where: $\beta$ is the angle, $\beta \in \left[ {0,\pi } \right]$, if $\beta$ takes the value $0$, $\pi /2$ and $\pi$, the position of this dung beetle is not updated when the value is taken.

(2) Breeding behavior

**Figure 1:**
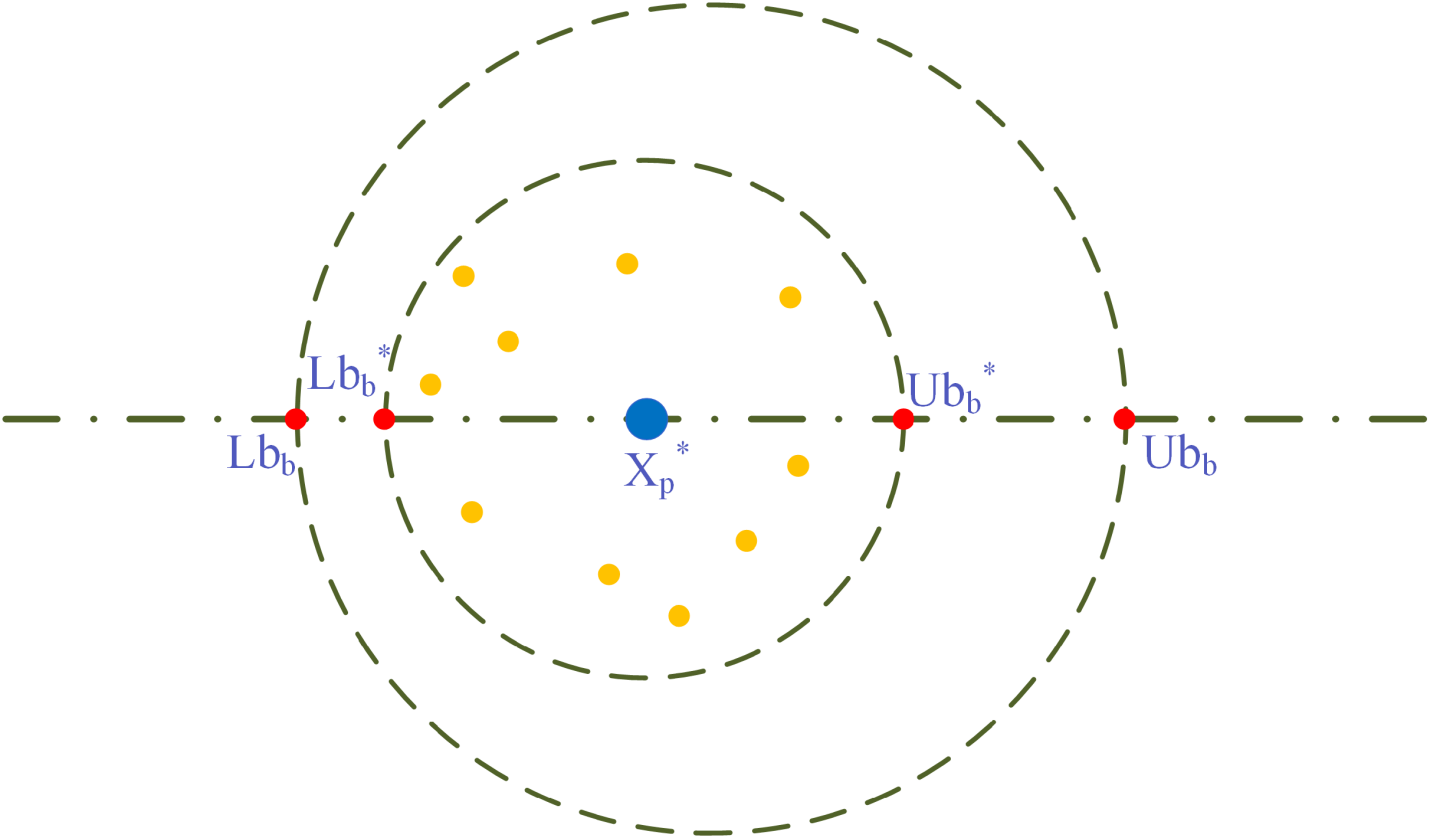
Model of spawning boundary selection area.

In the wild, dung beetles choose a secured area in which to lay their eggs, and to simulate this behavior; in order to simulate this behavior, a strategy of choosing a boundary to model the region is proposed; the equation for this boundary region is:

(3) $$\left\{ {\matrix{ {L{b_b}^ * = \max ({X_p}^ * (1 - M),L{b_b})}\hfill \cr \matrix{U{b_b}^ * = \min ({X_p}^ * (1 + M),U{b_b}) \hfill \cr M = {t_p}/{T_{\max }} \hfill} \cr } } \right.$$

where: $L{b_b}^ *$ and $U{b_b}^ *$ are considered bottom and upper boundaries for the egg-laying zone, $L{b_b}$ and $U{b_b}$ represent bottom and top bounds over the optimization problem, respectively, ${X_P}^ *$ is the current local best location, ${t_P}$ is the number of iterations for the current iteration, the number of the most prominent iterations is ${T_{\max }}$, and $M$ determines the dynamic change of the spawning area. The prevailing local best site ${X_p}^ *$ is represented with the large blue circle depicted in Fig. 1, and the little yellow colored circle surrounded by ${X_p}^ *$ represent the ovoid. Every ovoid will contain a dung beetle seed. In addition, the circles in red symbolize an upper and lower limit for the border.

As for the algorithm of DBO, each female dung beetle produces only a single egg in each iteration, and each egg-laying dung beetle spawn updates the location of the dung beetle. The area of spawning is variable and moving; thus, it is guaranteed to search the area with the current best solution and at the same time prevent falling into a partial optimal. Positional updating of egg-laying dung beetles is done in the following manner:

(4) $$\eqalign{ & {B_b}({t_p} + 1) = {X_p}^ * + {a_1} \times ({B_b}({t_p}) - L{b_b}^ * )  + {a_2} \times ({B_b}({t_p}) - U{b_b}^ * )}$$

where: ${B_b}({t_p})$ represents where the ${b_{}}th$ spawning sphere is located at the ${t_p}_{}th$ of iteration; ${a_1}$ and ${a_2}$ denote two random independent vectors with magnitude $1 \times D$, where $D$ represents the dimension of optimization.

(3) Foraging behavior

Some adult dung beetles will burrow out of the ground for their meals. They are small dung beetles. In addition, optimal foraging areas require the establishment of optimal foraging areas to lead them to seek foraging beetles, simulating the feeding habits of these dung beetles as they forage for food throughout the natural world. In particular, an equation for the boundary of foraging areas of the best foraging area is given by:

(5) $$\left\{ {\matrix{ {L{b_b} = \max ({X_g}^b(1 - M),L{b_b})}\hfill \cr \matrix{U{b_b} = \min ({X_g}^b(1 + M),U{b_b}) \hfill \cr M = {t_P}/{T_{\max }} \hfill} \cr } } \right.$$

where: $L{b_b}^b$ and $U{b_b}^b$ are the bottom and top boundaries of optimal foraging areas, $L{b_b}$ and $U{b_b}$ denote bottom and top bounds over the optimization problem, respectively, ${X_g}^b$ being the best location globally, ${t_p}$ is the number of iterations for the current iteration, the number of the most oversized iterations is ${T_{\max }}$, and $M$ determines the dynamic change of the optimal foraging area.

Small dung beetles update their position in the area where they are foraging by:

(6) $$\eqalign{ & {x_f}({t_p} + 1) = {x_f}({t_p}) + {D_1} \times \left( {{x_f}({t_p}) - L{b_b}^b} \right)  + {D_2} \times \left( {{x_f}({t_p}) - U{b_b}^b} \right)}$$

where: ${x_f}({t_p})$ represents the position of the *fth* baby dung beetle during the ${t_p}_{}th$ iteration, ${D_1}$ for arbitrary numbers obeying a regular partition, ${D_2} \in (0,1)$.

(4) Theft behavior

Some of these dung beetles, known as thieves, would be able to take balls of dung from others, ${X_b}^b$ denotes the best location to compete bite-size portion of food, and the positional information for stealing this is updated during the iteration process in the following way:

(7) $$\eqalign{ & {x_t}\left( {{t_p} + 1} \right) = {X_t}^b + F \times h  \times \left\{ {\left| {{x_t}\left( {{t_p}} \right) - {X_p}^ * } \right| + \left| {{x_t}\left( {{t_p}} \right) - {X_t}^b} \right|} \right\}}$$

where: ${x_t}\left( {{t_p}} \right)$ is one of the locations where the ${t_{}}th$ stealer is located during the ${t_p}_{}th$ iteration; $h$ denotes an arbitrary variable with $1 \times D$ dimensions and following a regular pattern; $F$ is a constant value.

### BP neural network

**Figure 2:**
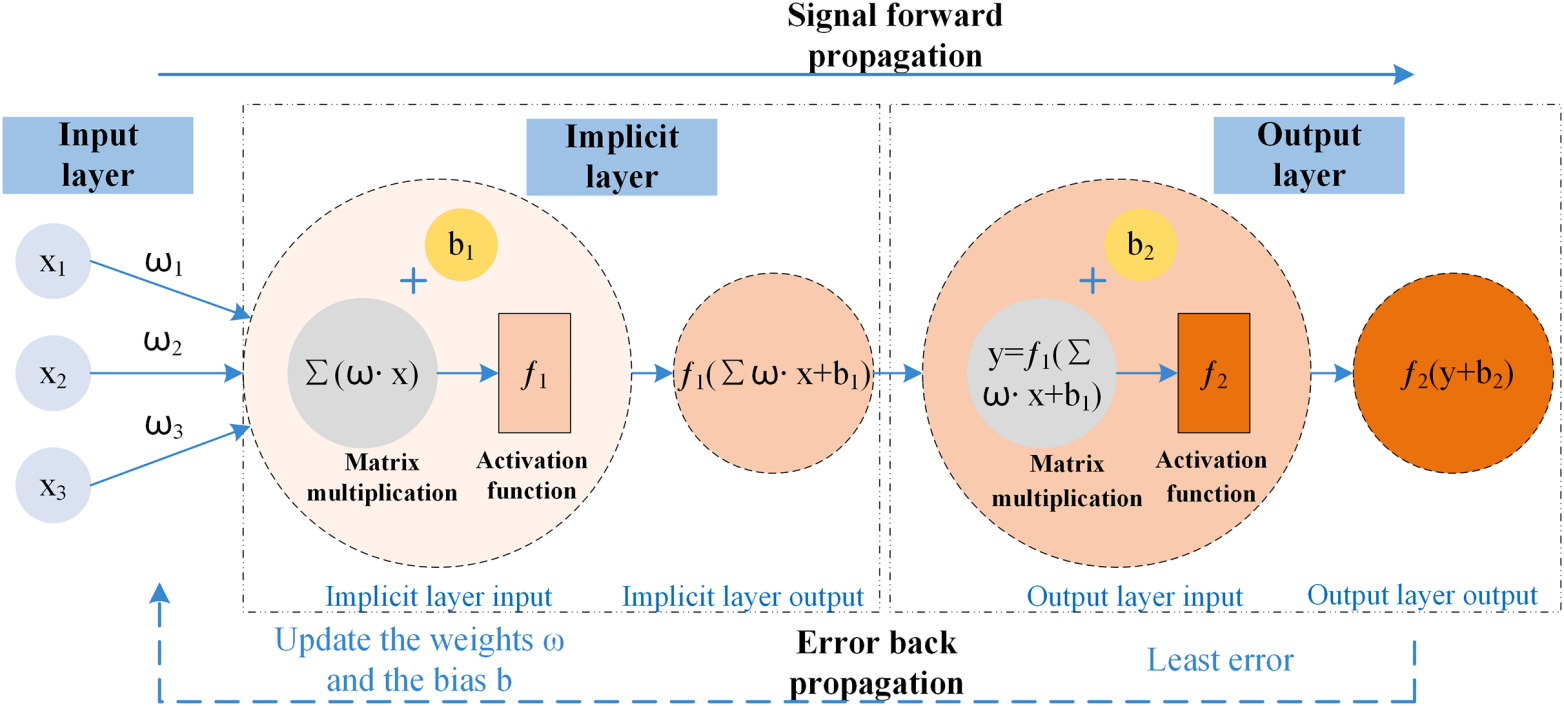
Schematic of BP neural network propagation.

BP neural network is a backpropagation algorithm for learning with multi-layer networks (Yan, 2015); it has mechanisms for both the input data propagating ahead and the error to be spread in reverse. The framework allows the mathematical projections to reflect the intrinsic laws of the acquired data, as well as a solid nonlinear fitting ability; the training process of the BP neural network is to optimize the weights and thresholds (He et al., 2023) of the prediction model so that the loss function reaches a minimum so that it can minimize the error of the output value concerning the actual value, to achieve the effect of approximating the effect of a variety of nonlinear continuous functions. The BP neural network has a two-stage working mechanism and is simultaneously divided into three parts: input, hidden and output layers; the propagation process consists of input node $x$, weights $w$, bias $b$, activation function $f$, output node $y$, BP neural network data is propagated ahead and the error is spread in reverse schematic shown in Fig. 2.

### The SLPDBO algorithm

While the DBO algorithm is characterized by high accuracy, high optimality finding performance, and high time of convergence, it also has the disadvantage of not being able to keep the two phases of the exploration and the development phases well-balanced, being easy to fall into the local optimum, and having the disadvantage of being less able to explore the whole picture. Therefore, in an attempt to boost the efficiency of the DBO finding property, the article proposes a multi-strategy algorithm to improve DBO. The merit-seeking capability of the DBO algorithm is improved by the sinusoidal chaotic mapping strategy, Levy flight strategy, and particle swarm optimization algorithm (PSO) fusing adaptive weights and variational operators. The DBO algorithm, which is improved by a mixture of multiple strategies, is called the SLPDBO algorithm. This chapter will specifically introduce these strategies.

#### Sinusoidal chaos mapping

**Figure 3:**
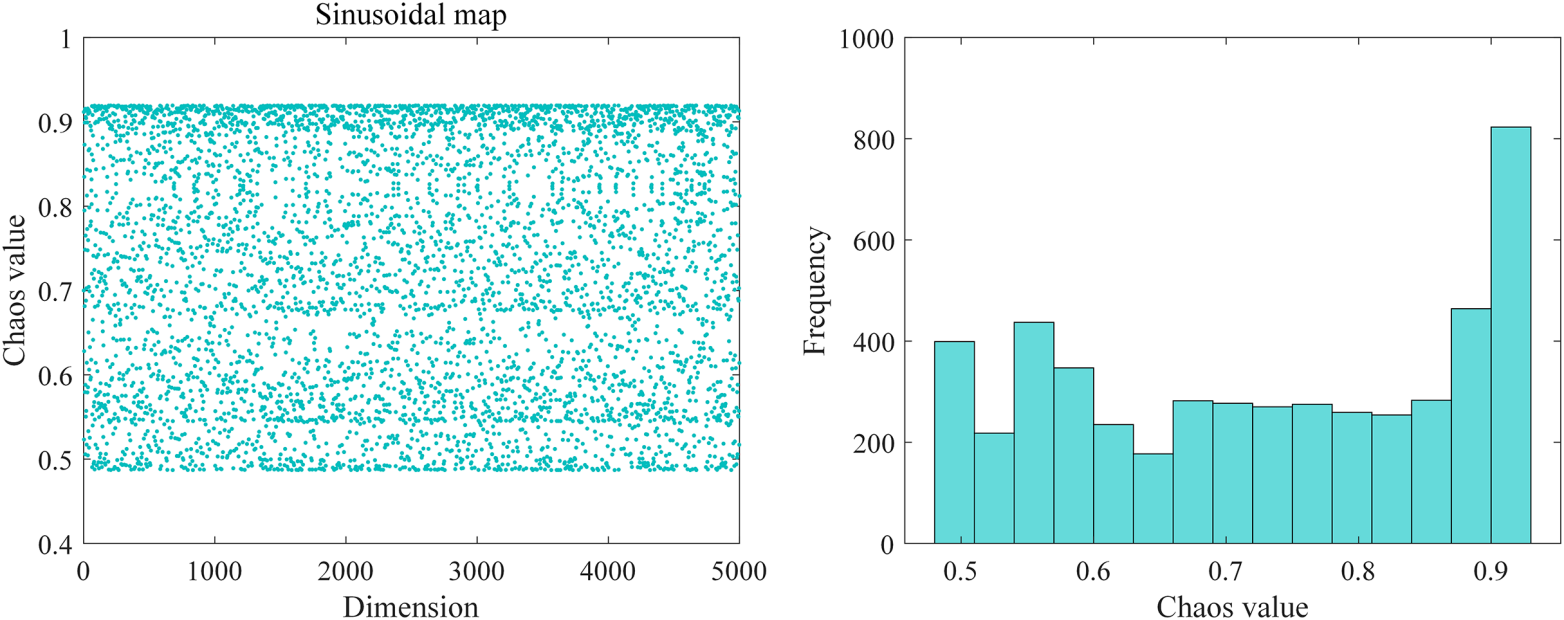
Sinusoidal chaotic mapping distribution and frequency map.

Chaos mapping is a randomness sequence generated by simple nonlinear, deterministic, ergodic, and stochastic properties of the perturbation mechanics system used to generate chaotic sequences (Toktas et al., 2023). The traditional DBO algorithm is to have a random seed to generate the initial population, so there a disadvantage of poor initial position, easy trapping in local optimization, *etc*., and the application of sinusoidal chaos mapping can be excellent to circumvent the search of the solution space, make the search field wider, increases the broadness breadth of the optimization alchemy, makes the original settlement as homogeneous as possible in the resolution space energy so that the convergence speed of the algorithm become faster. Figure 3 shows the sinusoidal chaotic mapping distribution and frequency diagram. Sinusoidal mapping is utilized to project the resulting values into chaotic variables. Then, the resulting chaotic values are mapped into the initial space of the algorithm to find the initial position using a linear transformation. As seen in the figure, the chaotic sequence generated by sinusoidal chaotic mapping is more uniform, making the position update traversal random, helping the algorithm to jump out of the local optimum and explore a wider solution space. Meanwhile, the regularity of the chaotic sequence helps the algorithm find potential solutions faster and accelerate the convergence process. The specific expression of sinusoidal mapping is:

(8) $${{\rm X}_{t + 1}} = {\rm aX}_t^2{\rm sin}(\pi {\rm X}_t^{})$$

where: ${{\rm X}_{\rm t}}$ is the current value of the chaotic sequence at the ${t_{}}th$ iteration; ${\rm a}$ is the control coefficient; in the article, the traversal works best when taken as 2.

#### Levy flight strategy

Levy flight is a stepwise probability-distributed stochastic nomadic maneuver (Joshi, 2023), predetermined from a likelihood distribution defined by the Levy allocation (Li et al., 2022). The movement of dung beetle individuals mainly relies on the location details of the most optimized individual to update; in the position update equation of dung beetle stealing behavior, when the optimal individual falls into a local optimal solution, the other individuals of the dung beetle population will fall into a stagnant state when they move towards the optimal individual. Levy flights have the characteristics of long step lengths and distances, long-tailed distributions, and stochastic movement patterns, so in this article, we use Levy flights to enhance the path disturbance when an individual moves towards the Therefore, this article uses Levy flight to enhance the path perturbation when the individual moves to the current optimal position, which contribute to a better algorithm for tripping out local optima solution and makes the algorithm have better diversity. Levy flight provides large-scale exploration in the initial phase and fine-grained exploitation in the later phase through short step sizes, thus balancing exploration and exploitation to find the global optimal solution by using Levy flight to perturb the location of the solution in the position update formulation in the stealing behavior, enhancing the global search capability. The position update equation after the Levy flight strategy perturbation is shown below:

(9) $$\eqalign{  {x_t}\left( {{t_p} + 1} \right) = {X_t}^b + F \times h  \times \left\{ {\left| {{x_t}\left( {{t_p}} \right) - {X_p}^ * } \right| + \left| {{x_t}\left( {{t_p}} \right) - {X_t}^b} \right|} \right\} \times \gamma \oplus Levy(\lambda )}$$

where: $\gamma$ indicates a random step, $\oplus$ means a dot product, $Levy(\lambda )$ denotes a randomized path of search and satisfying the constraint equation as:

(10) $$L{\rm evy}(\lambda ) \sim \mu = {t^{ - \lambda }},1 \; < \; \lambda,3$$

The Levy flight random step is generated by:

(11) $$s = \displaystyle{\mu \over {|\nu {|^{\textstyle{1 \over \beta }}}}}$$

where: $\mu$ and $\nu$ are parameters obeying a normal distribution, $\mu \sim N\left( {0,\sigma _\mu ^2} \right)$, $\nu \sim N\left( {0,\sigma _\nu ^2} \right)$. ${\sigma _\mu }$ and ${\sigma _v}$ are:

(12) $${\sigma _\mu } = {\left[ {\displaystyle{{{ \Gamma }\left( {1 + \beta } \right)\sin \left( {\pi \beta /2} \right)} \over {{ \Gamma }\left( {\left( {1 + \beta } \right)/2} \right)\beta {2^{\left( {\beta - 1} \right)/2}}}}} \right]^{\textstyle{1 \over \beta }}},\quad {\sigma _\nu } = 1$$

where: ${ \Gamma (x)}$ is the Gamma function; $\beta \in (0,2)$, the empirical value 1.5 is taken here.

#### PSO fusion adaptive weight mutation operator

PSO strategy comes from the position update formula of particle swarm optimization algorithm (Jiang et al., 2021), PSO algorithm is mainly updated by searching for the optimal speed and position of each particle, Taking into account that the dung beetle algorithm suffers from a tendency to fall into local optimality, the introduction of the PSO position update strategy jumps out of the local optimal, and improves the algorithm’s global search ability; simultaneously, the introduction of inertia weight, the nonlinear descending characteristics, gradually decreasing with the increase in the number of iteration decrease, can make the particles maintain inertial motion, increasing the scope of seeking out space and the ability to explore new regions, it improved the acceleration of the later phase of the integration of the method; increase the variation operator, the variation probability with the increase in the number of iterations and gradually reduce, help in the early exploration of the algorithm to discover more solvability space, and at a later stage, the refined search for the optimal solution finally realizes the algorithm’s improvement in convergence speed and optimality searching ability. The PSO algorithm position updating formula is:

(13) $$\eqalign{  {v_g}({t_p} + 1) = w{v_g}({t_p}) + b1 \cdot \epsilon 1 \cdot (p{X_g} - {x_g})  + b2 \cdot \epsilon 2 \cdot (bestX - {x_g})}$$

(14) $${x_g}({t_p} + 1) = {x_g}({t_p}) + {v_g}({t_p} + 1)$$

(15) $$w = {w_{\max }} - \left( {\displaystyle{{{w_{\max }} - {w_{\min }}} \over M}} \right) \cdot {t_p}$$

(16) $$P = 0.1 \cdot \left( {1 - \displaystyle{{{t_p}} \over M}} \right)$$

where: $w$ is the inertia weight, ${v_g}({t_p})$ is the velocity of the individual in the ${t_p}_{}th$ iteration. $\epsilon 1$ and $\epsilon 2$ are constants with values $0 \sim 1$, $b1$ and ${\rm b2}$ are acceleration factors of 1.5, $p{X_g}$ is the local optimum of the individual in the $t$ iteration, $bestX$ is the global optimum in the $t$ iteration, ${x_g}$ is the location of the individual in the ${t_p}_{}th$ iteration, $P$ represents the probability of variability concerning the number of iterations, ${w_{\max }}$ is taken to be 0.9, ${w_{\min }}$ is taken to be 0.4.

#### SLPDBO-BP neural network model construction

Aiming at the problems of solid subjectivity of traditional assessment methods, relying on manually formulated rules and indexes, inability to take full consideration of the sophisticated correlations among different types of data, insufficiently accurate and precise measurement of the value attributed to the data, and low evaluation efficiency, assessing the value of data assets using a BP neural network structure, which enables achieve efficient assessment and prediction of the asset value of data resources. Although more vital nonlinear mapping ability (Xue, Tong & Neri, 2022) self-learning and self-adaptation and generalization are excellent (Li, Li & Lian, 2015) features of BP neural networks, considering the disadvantages of BP neural networks that do not learn quickly, tend to fall into partial optimization and have low prediction accuracy. The excellent performance of the DBO algorithm is used to optimize the BP neural network weights and thresholds to achieve the minimum error, and finally, optimal results are presented. Aiming at the DBO algorithm’s problems in local extremes and convergence speed, the DBO algorithm is improved with a comprehensive strategy to establish a stable SLPDBO-BP prediction model and improve the prediction accuracy and generalization ability.

BP neural network is selected as the core model for data asset value assessment, mainly based on the following reasons: Firstly, nonlinear mapping ability; data asset value is affected by the nonlinearity of multiple factors, and the traditional linear model (*e.g*., regression analysis) is difficult to capture complex associations. BP neural network can fit the nonlinear relationship effectively through the multilayered neuron structure (input layer-implicit layer-output layer) and activation function. Secondly, self-learning and generalization ability; BP adjusts the parameters through the error back propagation mechanism, without relying on artificial rules, and can adapt itself to different data. Finally, it can fit with the needs of data asset assessment; data asset assessment needs to deal with the fusion of structured and unstructured data. BP neural network supports multi-source inputs, and there have been studies to validate its effectiveness in the field of asset assessment.

**Figure 4:**
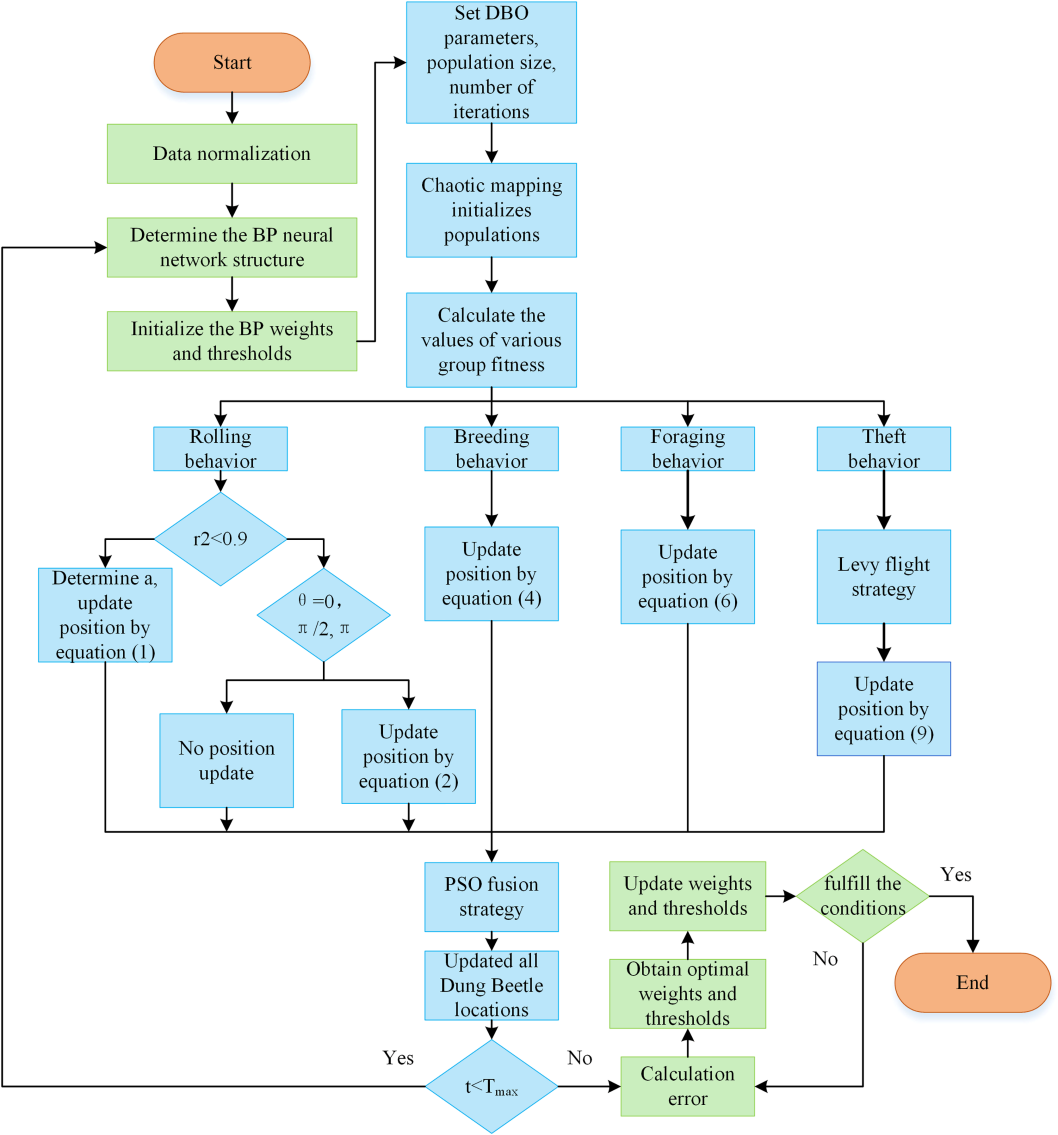
SLPDBO-BP model optimization flow chart.

The gradient descent method of BP is prone to falling into local optima, but this shortcoming can be compensated by intelligent optimization algorithms. This framework of BP fusion optimization algorithm has been widely used for complex problems such as wind power prediction, financial risk assessment, *etc*. and has proved its feasibility. The training objective of the BP neural network is to find the weights and thresholds that minimize the loss function. The traditional gradient descent method updates the parameters through local gradients, which is easy to fall into local optima and is slow to converge. SLPDBO, as a global optimization algorithm, is able to optimize the initial weights and thresholds of the BP by simulating the behavior of dung beetles and exploring the parameter space efficiently by using a search strategy that can jump out of local extremes. By finding better initial parameters through global search, the loss function surface of BP is closer to the global optimum. Compared with the existing methods, the proposed method has a strong nonlinear processing capability and is suitable for dealing with complex, nonlinear data asset value assessment. Especially in the case of large data volumes and many influencing factors, the performance is better. Traditional types of methods, such as the cost method, income method, and market method, are simple to operate. Considering the historical cost may ignore future income, as well as market and other circumstances, is a limitation. They are more subjective and easily affected by fixed thinking, and their scope of use is more limited. Therefore, we chose to use the SLPDBO-BP model for the assessment of the value of data assets. The steps for the specific optimization are illustrated below:

(1) The data was analyzed and processed to build a BP neural network, determining the network’s input and output structure and initialization of the linking weights and thresholds of the BP neural network.
(2) Initialize the improved DBO parameters, calculate the DBO algorithm’s decision length, and set the initial conditions of the DBO, including setting the population dimensions, the maximum iteration number, spatial dimensions, boundaries, and so on. Meanwhile, the mean square error (MSE) is selected as the objective function of the improved DBO-optimized BP.
(3) Chaotic mapping initializes the population.
(4) Calculating individual fitness in dung beetle populations.
(5) The position is constantly updated according to Eqs. (1), (2), (4), (6), (9).
(6) Determine if the dung beetle is out of bounds after each update.
(7) First of all, we calculate the individual fitness value. Then, we find the optimum fitness value location, record the location vector, and use it as the optimal individual location.
(8) The algorithm stopping criterion is set to stop the algorithm by terminating the optimization process after the maximum number of iterations is satisfied or the error accuracy requirement is reached. The optimized optimal weight threshold parameter of the improved DBO algorithm is given to the BP neural network; that is, the optimal SLPDBO-BP model is outputted. As depicted in Fig. 4, the SLPDBO-BP model optimization process is demonstrated.

### Complexity analysis

When improving the underlying DBO algorithm, time complexity (Alkan & Bullock, 2021) being as follows factor to be considered, which is designed to represent the trend of code execution time as the data size increases. Time complexity describes the execution duration of an algorithm under the worst possible time, indicating the time resources needed to run it. It is used to compare the efficiency of different algorithms. Time complexity can be expressed in terms of Big-O representation (Pelusi et al., 2018) as a measure of how good an algorithm is.

We hypothesize that *N* indicates the village size, D denotes the optimization problem vector, and M states the maximum number of iterations. The time complexity analysis is performed for the DBO algorithm, and the synthesis strategy improves the algorithm SLPDBO. The temporal sophistication of the initial segment has been M1 = *O*(*N* * *D*), and N dung beetles undergo a position update in each iteration, iterating M rounds; the intricate degree of the alternation process is M2 = *O*(M * N * D), so the iterative process has the complexity of M1 + M2 simplified to *O*(M * N * D) for the DBO algorithm. The initialization phase by the SLPDBO algorithm has the same intricate complexity as the DBO algorithm as M1, M rounds of iterations in which SLPDBO improves the DBO stealing behavior assuming that the share of stolen dung beetles is P, M3 = *O*(M * P * *D*), and positional updating of the entire species of dung beetles using the PSO fusion strategy, M4 = *O*(M * N * D), therefore the sophistication of the SLPDBO alphabet is M1 + M3 + M4 simplified to *O*(M * N * D). In summary, the SLPDBO has a complexity profile identical to that of the DBO. The efficiency of the improved algorithm has not decreased.

## Experimental tests and analysis of results

### Experimental setup

The aim is to objectively evaluate the SLPDBO algorithm’s optimization-seeking ability and the effectiveness of the integrated strategy. When selecting test functions, it is essential to ensure that the chosen function comprehensively evaluates the performance of the algorithm in different contexts, highlighting the assessment of convergence ability, handling of complexity, local *vs*. global optimization, and adaptability in terms of dimensionality. In order to verify the convergence speed and efficiency of the algorithm, 20 commonly used standardized test functions have been selected for experimental simulation in this article, in which the functions ${F_1} - {F_4}$ are unimodal functions. There is a single solution that is globally optimal, which is designed to benchmark the acceleration of convergence and the ability of the algorithm to find an optimum. ${F_5} - {F_{10}}$ are simple multimodal functions containing several partially optimal solutions and just one generally optimal resolution, which is employed to measure the capability of the algorithm for national selection and mining. ${F_{11}}$, ${F_{12}}$, ${F_{13}}$, and ${F_{14}}$ are composite modal benchmark test functions due to their more complex composition with additional bias values and a weight for each subfunction. These complex combinatorial functions which directly add further challenge and time required to find the optimal point of the algorithm. Multiple local minima in the interval belonging to the algorithm are often used to test the algorithm’s ability to balance the relationship between global search and local development. ${F_{15}} - {F_{20}}$ are fixed dimensional modal functions used to manage the performance of a specific algorithm or data structure in different dimensions. In Table 1 the mathematical expressions, the optimization search dimension, the range of values and the minimum value of the corresponding 20 benchmark functions are shown.

**Table 1:** A total of 20 benchmark functions.

Function type	Displayed formula	Dimensions	Search range	Optimum value
Unimodal functions	${F_1}(x) = \sum \limits_{i = 1}^D {({10^6})^{{{i - 1} \over {D - 1}}}}z_i^2 + f_1^ * ,z = {T_{osz}}({{\bf{M}}_1}(x - o))$	30	[−100,100]	−1,300
	${F_2}(x) = z_1^2 + {10^6}\mathop \sum \limits_{i = 2}^D z_i^2 + f_2^ * ,z = {{\bf{M}}_2}T_{asy}^{0.5}({{\bf{M}}_1}(x - o))$	30	[−100,100]	−1,200
	${F_3}(x) = \mathop \sum \limits_{i = 1}^D {({10^6})^{{{i - 1} \over {D - 1}}}}x_i^2$	30	[−100,100]	100
	${F_4}(x) = {10^6}x_1^2 + \mathop \sum \limits_{i = 2}^D x_i^2$	30	[−100,100]	300
Simple multimodal functions	${F_5}(x) = - 20\exp \left( - 0.2\sqrt {{1 \over D}\mathop \sum \limits_{i = 1}^D x_i^2}\, \right) - \exp \left({1 \over D}\mathop \sum \limits_{i = 1}^D \cos (2\pi {x_i})\right) + 20 + e$	30	[−100,100]	500
	${F_6}(x) = \mathop \sum \limits_{i = 1}^D (x_i^2 - 10\cos (2\pi {x_i}) + 10)$	30	[−100,100]	800
	${F_7}(x) = {{10} \over {{D^2}}}\mathop \prod \limits_{i = 1}^D {\left(1 + i\mathop \sum \limits_{j = 1}^{32} {{\left| {{2^j}{x_i} - round({2^j}{x_i})} \right|} \over {{2^j}}}\right)^{{{10} \over {{D^{1,2}}}}}} - {{10} \over {{D^2}}}$	30	[−100,100]	1,000
	${F_8}(x) = {\left| {\mathop \sum \limits_{i = 1}^D x_i^2 - D} \right|^{1/4}} + \left(0.5\mathop \sum \limits_{i = 1}^D x_i^2 + \mathop \sum \limits_{i = 1}^D {x_i}\right)/D + 0.5$	30	[−100,100]	1,100
	${F_9}(x) = {\left| {{{\left(\mathop \sum \limits_{i = 1}^D x_i^2\right)}^2} - {{\left(\mathop \sum \limits_{i = 1}^D {x_i}\right)}^2}} \right|^{1/2}} + \left(0.5\mathop \sum \limits_{i = 1}^D x_i^2 + \mathop \sum \limits_{i = 1}^D {x_i}\right)/D + 0.5$	30	[−100,100]	1,200
	$\eqalign{ & {F_{10}}(x) = {\sin ^2}\left( {\pi {w_1}} \right) + \mathop \sum \limits_{i = 1}^{D - 1} {\left( {{w_i} - 1} \right)^2} \\ & \times \left[ {1 + 10{{\sin }^2}\left( {\pi {w_i} + 1} \right)} \right] + {\left( {{w_D} - 1} \right)^2}\left[ {1 + {{\sin }^2}\left( {2\pi {w_D}} \right)} \right] \cr & {w_i} = 1 + {{{x_i} - 1} \over 4},\forall i = 1, \ldots ,D}$	30	[−100,100]	900
Composition functions	${F_{11}}(x) = F(x) = \mathop \sum \limits_{i = 1}^N \{ {\omega _i} * [{\lambda _i}{g_i}(x) + bia{s_i}]\} + F *$Composition Function 4 (*N* = 5)	30	[−100,100]	2,600
	${F_{12}}(x) = F(x) = \mathop \sum \limits_{i = 1}^N \{ {\omega _i} * [{\lambda _i}{g_i}(x) + bia{s_i}]\} + F *$Composition Function 6 (*N* = 5)	30	[−100,100]	2,800
	${F_{13}}(x) = F(x) = \mathop \sum \limits_{i = 1}^N \{ {\omega _i} * [{\lambda _i}{g_i}(x) + bia{s_i}]\} + F *$Composition Function 4 (*N* = 4)	30	[−100,100]	2,300
	${F_{14}}(x) = F(x) = \mathop \sum \limits_{i = 1}^N \{ {\omega _i} * [{\lambda _i}{g_i}(x) + bia{s_i}]\} + F *$Composition Function 8 (*N* = 6)	30	[−100,100]	2,700
Fixed dimension functions	${F_{15}}(x) = {\left| {\mathop \sum \limits_{i = 1}^D x_i^2 - D} \right|^{1/4}} + \left( {0.5\mathop \sum \limits_{i = 1}^D x_i^2 + \mathop \sum \limits_{i = 1}^D {x_i}} \right)/D + 0.5$	10	[−100,100]	1
	${F_{16}}(x) = \mathop \sum \limits_{i = 1}^n - {x_i}\sin \left(\sqrt {|{x_i}|} \right)$	30	[−500,500]^n^	−12,569.5
	${F_{17}}(x) = {\left({x_2} - \displaystyle{{5.1} \over {4{\pi ^2}}}x_1^2 + \displaystyle{5 \over \pi }{x_1} - 6\right)^2} + 10 \left(1 - \displaystyle{1 \over {8\pi }}\right)\cos {x_1} + 10$	2	[−5,10]*[0,15]	0.397887
	$\eqalign{ {F_{18}}(x) = \; & [1 + {({x_1} + {x_2} + 1)^2}(19 - 14{x_1} + 3x_1^2 - 14{x_2} + 6{x_1}{x_2} + 3x_2^2)] \cr & \times [30 + {(2{x_1} - 3{x_2})^2}(18 - 32{x_1} + 12x_1^2 + 48{x_2} - 36{x_1}{x_2} + 27x_2^2)]}$	2	[−2,2]^D^	2.999999
	${F_{19}}(x) = - \mathop \sum \limits_{i = 1}^4 {c_i}\exp \left( - \mathop \sum \limits_{j = 1}^3 {a_{ij}}{({x_j} - {p_{ij}})^2}\right)$	3	[0,1]^D^	−3.862782
	${F_{20}}(x) = - \mathop \sum \limits_{i = 1}^4 {c_i}\exp \left( - \mathop \sum \limits_{j = 1}^6 {a_{ij}}{({x_j} - {p_{ij}})^2}\right)$	6	[0,1]^D^	−3.321995

For the purpose of testing the optimization-seeking capability of the SLPDBO algorithm, firstly, comparison experiments are conducted with different intelligent optimization algorithms, Genetic Algorithm (GA) (FuRui, Al-Absi & Lee, 2019), Whale Optimization Algorithm (WOA) (Mirjalili & Lewis, 2016), Subtractive Averaging of Optimization Algorithm (SABO) (Trojovsky & Dehghani, 2023), Golden Jackal Optimization Algorithm (GJO) (Chopra & Ansari, 2022), Chimp Optimization Algorithm (ChOA) (Khishe & Mosavi, 2020) and the DBO algorithm. They responded to the algorithm’s convergence accuracy and stability by comparing the mean, standard and optimal values of the fitness values in the 20 functions, which finally verifies the superiority of the SLPDBO reaction algorithm. Secondly, ablation experiments with algorithms with different improvement strategies are conducted to verify the superiority of the SLPDBO algorithm in terms of comprehensive performance. In the ablation experiments, the algorithm based on the sinusoidal chaos mapping strategy is SDBO, the algorithm based on the Levy flight strategy is LDBO, and the algorithm based on the PSO fusion adaptive weight variational operator is PDBO. Table 2 shows the corresponding parameter settings for the selected algorithms. These parameters can be adjusted accordingly for different applications.

**Table 2:** Algorithm parameter settings.

Algorithm	Parameters	Value
GA	pc, pm	0.8, 0.05
WOA	A, C, b, l, a, a2, r1, r2, p	2 * a * r1–a, C = 2 * r2, 1, [−1,1], [0,2], [−1, −2], [0,1], [0,1], [0,1]
SABO	I, r	[0,2], [0,1]
GJO	RL, c1, E0, E1, u, v, β	0.05 * Levy(y),1.5, [−1,1] [0,1.5], [0,1], [0,1],1.5
ChOA	f, r1, r2, a, m	[0,2.5], [0,1], [0,1], [−2f,2f], chaos (3)
DBO	b, k, a, θ, R, RDB, EDB, FDB, SDB	0.3, 0.1, 1 or −1, [0, π], 1–t/M, 6, 6, 7, 11
SLPDBO	b, k, a, θ, R, RDB, EDB, FDB, SDB sinusoidal, RL, w-max, w-min	0.3, 0.1, 1 or −1, [0, π], 1−t/M, 6, 6, 7, 11, 2, RL = 0.15 * Levy(y), 0.9, 0.4

In this article, the core hyperparameters are determined based on practical experience and hierarchical progressive parameter adjustment method. In the SLPDBO optimization algorithm, in the selection of population size, the three sizes of [20,30,50] are tested by the control variable method, and it is found that there is no significant improvement in convergence stability of the algorithm when the size is larger than 30, so the population size is selected to be 30 to balance the efficiency and accuracy. In the sinusoidal chaotic mapping coefficients according to reference (Ali et al., 2023) is set to $\mu = 2.3$, which can ensure the chaotic sequence ergodicity. Levy flight step, scaling factor for $\lambda$, $\lambda \in [1,3]$ when the global exploration ability is optimal, and finally take 1.5.

### Test function experimental analysis

The test in this article operates on the Windows 11 Professional Edition operating system. Meanwhile, the processor of this computer is Intel(R) Core (TM) i7-10700, clocked at 2.90 GHz, the memory is 8 GB, and MATLAB2022b is used for coding. The initial parameters for the setup of this article are that each alternative algorithm was run automatically for 30 cycles with the population set to 30 and a total maximum number of iterations of 500.

#### Test function test comparison

In order to verify the superiority and stability of the SLPDBO algorithm’s optimization, seeking the best with different intelligent optimization algorithms, as displayed in the outcome of the measurement experiments in Table 3, where bold indicates the optimal results. It is found that SLPDBO achieves the best results in the standard deviation of each function in the unimodal functions ${F_1} - {F_4}$, and achieves the optimal values in terms of accuracy and stability, as well as the average values in ${F_3}$ and ${F_4}$. In the optimal values, the minimum value of SLPDBO is higher than the GJO algorithm, which indicates that the performance is slightly lower than GJO. However, it is among the top of several algorithms. The proposed algorithm SLPDBO has superior results regarding local development capability, and its overall functionality performs more nicely than the others in terms of execution capability. In the optimal values, SLPDBO is slightly lower than the GJO algorithm but is among the top of several algorithms. The proposed SLPDBO algorithm has superior results in terms of local development capability, and its overall performance is better than that of the other algorithms in terms of execution capability. Among the multi-peak functions ${F_5}$– ${F_{10}}$, functions ${F_7}$ and ${F_8}$ have the best accuracy and stability in the mean and standard deviation.

**Table 3:** The experimental results of GA, WOA, SABO, ChOA, DBO and SLPDBO based on benchmark functions.

Function	Index	GA	WOA	SABO	GJO	ChOA	DBO	SLPDBO
F1	Best	1.272E+08	9.346E+07	8.725E+07	3.315E+07	7.293E+07	**2.066E+07**	7.985E+07
	Average	4.952E+08	1.769E+08	2.238E+08	**8.500E+07**	1.498E+08	1.056E+08	8.761E+07
	Standard	2.371E+08	2.287E+07	1.287E+08	5.170E+07	7.012E+07	2.012E+07	**1.098E+07**
F2	Best	5.498E+10	3.260E+10	2.439E+10	**1.386E+10**	4.144E+10	2.200E+10	2.302E+10
	Average	1.233E+11	4.589E+11	8.701E+12	**3.595E+10**	7.999E+12	1.654E+11	4.571E+10
	Standard	1.183E+10	4.839E+11	1.223E+13	2.746E+10	6.911E+12	1.531E+11	**4.441E+09**
F3	Best	6.972E+08	2.114E+08	2.242E+08	1.744E+08	4.724E+08	**8.956E+07**	1.237E+08
	Average	8.362E+08	2.406E+08	3.882E+08	3.079E+08	6.045E+08	1.906E+08	**1.376E+08**
	Standard	1.966E+08	4.136E+07	2.318E+08	1.889E+08	1.868E+08	1.430E+08	**1.963E+07**
F4	Best	2.311E+05	3.097E+05	5.977E+04	**4.518E+04**	9.090E+04	4.609E+04	5.225E+04
	Average	2.519E+05	3.122E+05	6.208E+04	5.977E+04	9.283E+04	5.673E+04	**5.388E+04**
	Standard	2.935E+04	3.437E+03	3.261E+03	2.063E+04	2.719E+03	1.506E+04	**2.304E+03**
F5	Best	5.210E+02	5.206E+02	5.209E+02	5.210E+02	5.209E+02	5.208E+02	**5.204E+02**
	Average	5.212E+02	5.209E+02	5.211E+02	5.211E+02	5.211E+02	5.210E+02	**5.208E+02**
	Standard	6.295E−02	1.031E−01	7.971E−02	**5.070E−02**	6.628E−02	9.750E−02	1.761E−01
F6	Best	1.232E+03	1.020E+03	1.068E+03	9.901E+02	1.036E+03	**9.501E+02**	9.579E+02
	Average	1.260E+03	1.072E+03	1.076E+03	9.980E+02	1.059E+03	9.821E+02	**9.705E+02**
	Standard	3.945E+01	7.350E+01	1.132E+01	**1.113E+01**	3.251E+01	4.518E+01	1.784E+01
F7	Best	6.237E+03	4.393E+03	7.028E+03	3.636E+03	6.624E+03	3.502E+03	**2.171E+03**
	Average	8.167E+03	6.310E+03	8.373E+03	5.904E+03	7.862E+03	4.903E+03	**4.208E+03**
	Standard	9.389E+02	7.838E+02	**5.210E+02**	1.436E+03	7.659E+02	8.671E+02	7.562E+02
F8	Best	7.477E+03	7.308E+03	9.086E+03	**5.279E+03**	8.847E+03	5.865E+03	5.928E+03
	Average	7.580E+03	7.350E+03	9.335E+03	7.122E+03	8.988E+03	7.117E+03	**6.402E+03**
	Standard	1.461E+02	**5.922E+01**	3.519E+02	2.606E+03	1.998E+02	1.771E+03	6.707E+02
F9	Best	1.203E+03	1.201E+03	1.202E+03	1.201E+03	1.202E+03	1.201E+03	**1.201E+03**
	Average	1.204E+03	1.202E+03	1.203E+03	1.203E+03	1.203E+03	1.203E+03	**1.202E+03**
	Standard	6.925E−01	5.714E−01	4.563E−01	8.248E−01	**4.546E−01**	9.950E−01	8.084E−01
F10	Best	1.090E+04	6.807E+03	7.300E+03	**4.836E+03**	1.225E+04	9.248E+03	5.808E+03
	Average	1.278E+04	1.091E+04	9.658E+03	7.708E+03	1.252E+04	9.545E+03	**6.548E+03**
	Standard	2.654E+03	5.801E+03	3.334E+03	4.061E+03	**3.806E+02**	4.208E+02	1.045E+03
F11	Best	2.711E+03	2.700E+03	2.709E+03	2.701E+03	2.704E+03	2.700E+03	**2.700E+03**
	Average	2.883E+03	2.731E+03	2.772E+03	2.764E+03	2.804E+03	2.714E+03	**2.701E+03**
	Standard	8.324E+01	6.363E+01	4.011E+01	4.757E+01	9.343E+01	3.442E+01	**2.012E−01**
F12	Best	6.974E+03	4.546E+03	4.671E+03	4.337E+03	5.392E+03	3.955E+03	**3.000E+03**
	Average	9.171E+03	5.854E+03	7.699E+03	5.194E+03	6.044E+03	4.849E+03	**4.498E+03**
	Standard	1.325E+03	7.481E+02	1.768E+03	8.748E+02	**3.325E+02**	5.005E+02	9.120E+02
F13	Best	4.349E+03	3.090E+03	3.408E+03	3.012E+03	3.257E+03	3.041E+03	**2.500E+03**
	Average	5.497E+03	3.433E+03	4.054E+03	3.298E+03	3.809E+03	3.292E+03	**3.008E+03**
	Standard	6.683E+02	2.065E+02	3.231E+02	**1.652E+02**	2.251E+02	1.904E+02	4.251E+02
F14	Best	5.649E+03	3.762E+03	3.789E+03	3.662E+03	4.061E+03	3.695E+03	**2.900E+03**
	Average	6.686E+03	4.139E+03	4.381E+03	4.448E+03	4.409E+03	3.954E+03	**3.753E+03**
	Standard	6.162E+02	2.338E+02	4.673E+02	3.420E+02	2.283E+02	**1.840E+02**	3.635E+02
F15	Best	1.271E+01	1.071E+01	1.072E+01	1.071E+01	1.073E+01	1.071E+01	**1.071E+01**
	Average	1.368E+01	1.175E+01	1.191E+01	1.232E+01	1.148E+01	1.119E+01	**1.105E+01**
	Standard	**1.823E−01**	9.639E−01	8.364E−01	8.090E−01	2.799E−01	4.799E−01	5.125E−01
F16	Best	−3.267E+03	**−1.257E+04**	−3.764E+03	−6.290E+03	−5.883E+03	−1.187E+04	−1.254E+04
	Average	−2.181E+03	−1.031E+04	−3.074E+03	−4.808E+03	−5.716E+03	−8.570E+03	**−1.063E+04**
	Standard	5.538E+02	1.760E+03	2.988E+02	9.561E+02	**6.742E+01**	1.742E+03	1.612E+03
F17	Best	6.198E+01	3.979E−01	3.979E−01	3.979E−01	3.979E−01	3.979E−01	**3.979E−01**
	Average	7.332E+01	3.979E−01	4.636E−01	3.979E−01	3.988E−01	3.979E−01	**3.979E−01**
	Standard	7.232E+00	6.159E−05	1.289E−01	1.840E−04	1.185E−03	**0.000E+00**	6.274E−07
F18	Best	3.000E+00	3.000E+00	3.001E+00	3.000E+00	3.000E+00	3.000E+00	**3.000E+00**
	Average	1.225E+01	3.000E+00	3.630E+00	3.000E+00	3.000E+00	3.000E+00	**3.000E+00**
	Standard	1.813E+01	3.187E−04	1.455E+00	3.122E−06	1.782E−04	**2.580E−15**	2.699E−04
F19	Best	−3.851E+00	−3.863E+00	−3.862E+00	−3.863E+00	−3.863E+00	−3.863E+00	**−3.863E+00**
	Average	−3.303E+00	−3.855E+00	−3.697E+00	−3.860E+00	−3.855E+00	−3.863E+00	**−3.863E+00**
	Standard	3.500E−01	1.208E−02	1.237E−01	3.627E-03	2.525E−03	1.439E−03	**3.544E−05**
F20	Best	−2.766E+00	−3.321E+00	−3.321E+00	−3.322E+00	−3.246E+00	−3.322E+00	**−3.322E+00**
	Average	−1.449E+00	−3.207E+00	**−3.265E+00**	−3.125E+00	−2.641E+00	−3.214E+00	−3.240E+00
	Standard	5.440E−01	1.023E−01	9.112E−02	2.005E−01	4.849E−01	1.516E-01	**6.051E−02**

**Note:**

Bold indicates optimal results.

In contrast, the standard deviation of the other functions achieves the optimal value, which is slightly lower than that of GJO and SABO in terms of the optimal value but is ranked at the top of several algorithms. Evidently, the SLPDBO algorithm has better exploratory competence in finding the optimal solution of a multi-peak function, the performance of the improved algorithm performs better than some well-known algorithms on most functions. In the composite functions ${F_{11}}$– ${F_{14}}$, all functions achieve the minimum in the optimum and standard deviation and are slightly less effective than the other algorithms in the standard deviation but also rank high among several algorithms. This shows the stabilizing effect of the SLPDBO algorithm on the search for excellence and its superior performance in balancing the ability of both the global seeking and the development of local relationships. In the fixed dimensional function ${F_{15}}$– ${F_{20}}$, ${F_{15}}$, ${F_{17}}$, ${F_{18}}$ and ${F_{19}}$ achieve the smallest value in the optimal value and the average value, ${F_{16}}$, ${F_{19}}$ and ${F_{20}}$ achieve the smallest value in the standard deviation, and the other functions of the indexes, although slightly lower, for example, the average value of ${F_{20}}$ is larger than the SABO algorithm, it means that the performance is lacking in this function. However, it is also ranked at the top of the algorithms.

**Figure 5:**
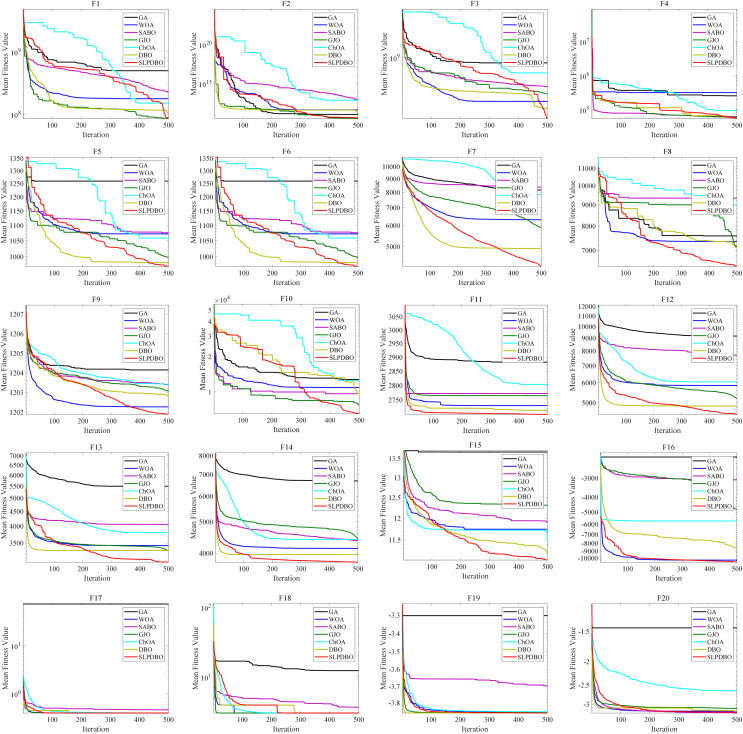
The convergence curves of SLPDBO and other algorithms on the benchmark functions.

Meanwhile, the seven comparison algorithms were iterated on 20 benchmark test functions, and the convergence course of the different algorithms is demonstrated in Fig. 5, where the number of iterations is demonstrated on the horizontal coordinate and the fitness value is demonstrated on the vertical coordinate. In most test functions, such as ${F_2}$, ${F_4}$, ${F_{11}}$– ${F_{20}}$, the SLPDBO algorithm has the fastest convergence speed and the highest convergence accuracy. The convergence curve approximates a straight-line convergence, which can converge and find the optimal value at the fastest speed. As can be discerned in ${F_1}$, ${F_3}$, and ${F_5}$– ${F_{10}}$ the SLPDBO algorithm maintains the fastest convergence speed, has the highest convergence accuracy, and converges with an approximately straight line convergence curve, and ultimately realizes the effect of converging at the fastest speed and finding the optimal value. In summary, no matter in the unimodal function, simple multimodal function, composite function, or fixed-dimension function, SLPDBO’s comprehensive performance is better than other standard algorithms, with superior performance in local exploitation and global exploration capabilities, and outstanding results in convergence of the accuracy and velocity of the optimization search. The superior performance of the improved SLPDBO algorithm in convergence optimization search is proved through comparative experiments, which is substantial to improve the convergence effect, exploration and development of the algorithm.

The performance advantage of SLPDBO over other algorithms when performing performance comparison experiments can be attributed to the differences in design between its core mechanism and the comparison algorithms. In the unimodal functions, SLPDBO ensures population diversity through sinusoidal chaotic mapping initialization by using an effective combination of various strategies to find a way to explore and develop global and local optima during initial position generation and position updating to avoid falling into local optima, which would lead to a reduction in the overall performance of the algorithm. In the multi-peak functions, SABO relies on random direction vectors for global exploration and lacks a guiding mechanism for the historical optimal solution, which causes it to easily fall into ineffective searches in complex multi-peak scenarios. The balancing factor of SABO is based on the adjustment of a fixed threshold, which makes it easy to terminate the search process prematurely when the region of local extremes is wider. In the composite functions, the step decay coefficient of GJO and the balance factor of SABO are fixed values, which are difficult to adapt to the differentiated search phase requirements in the composite function. The SLPDBO adaptively switches the global exploration according to the iterative progress to ensure that the optimal solution is well discovered. In the fixed-dimensional functions, such as GA and WOA, these algorithms are not as strong as SLPDBO in terms of adaptability, cannot be well adapted dynamically according to different problems, and search based on a fixed strategy, which cannot jump out of the local optimum. Ultimately, the effective combination of each strategy makes SLPDBO achieve superior performance in searching for the optimum.

#### Strategy effectiveness analysis

**Figure 6:**
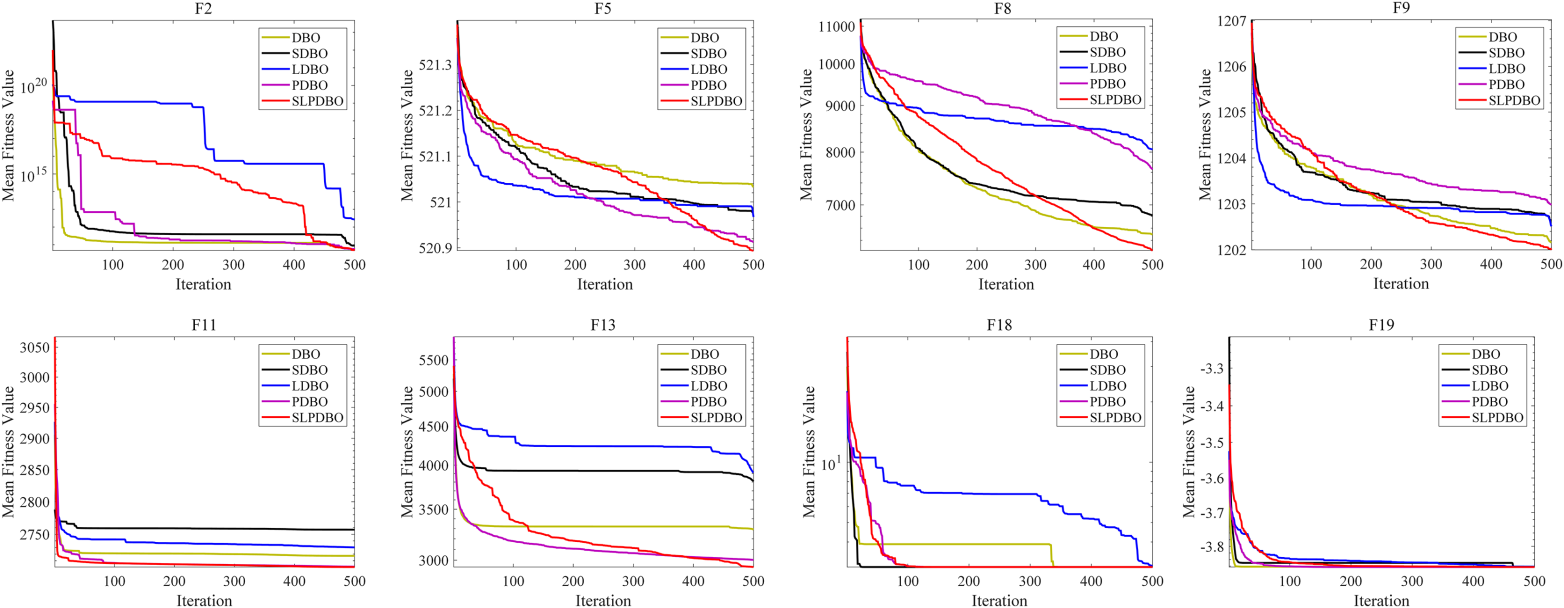
The convergence curves of SLPDBO and other DBO variants on the benchmark functions.

Different strategies enhance the performance of the algorithm in different ways; in an attempt to demonstrate the effectiveness of the combined revamp method, this research compares the SLPDBO algorithm with the original DBO algorithm with the integrated strategy improvement, the SDBO algorithm based on sinusoidal chaos mapping strategy, the LDBO algorithm based on the Levy flight strategy, and the PDBO algorithm based on the PSO fusion adaptive weight variational operator, as demonstrated in Table 4, the outcomes of the test and experiments for the comparison of different strategies are shown, where the bold denotes the optimal results. The convergence process is shown in Fig. 6. Analyzing the results and convergence diagrams, it is found that the SDBO algorithm and the original DBO are compared. However, the convergence curves are similar, and other results are the same; the optimal value of the SDBO method achieves the smallest value in the ${F_8}$, which indicates that the SDBO has improved the estimation accuracy to a certain extent. The generalized optimization hunting performance is getting more reliable. It is shown that introducing a sinusoidal statistical theory of chaotic mapping improves the quality and heterogeneity of the population while accelerating the convergence rate to generate a highly diverse initial population of dung beetles. Secondly, comparing the LDBO algorithm with the native DBO methodology, the standard deviation is minimized in ${F_5}$ and ${F_9}$, indicating that introducing the Levy flight strategy facilitates the algorithm to leapfrog from the partial minimum. The average optimization accuracy and speed can be further improved, balancing the capabilities of global discovery and also of local utilization of the algorithms. Finally, comparing the PDBO algorithm and the original DBO algorithm, the standard deviation in ${F_{13}}$ and ${F_{19}}$ obtains the minimum value, and the multiple values are more effective than the initially proposed algorithm, henceforth indicating that the introduction of PSO fusion adaptive weight variation operator strategy has a substantial increase in convergence speed and accuracy. It can dramatically improve the algorithm’s development ability, effectively avoiding the encroachment of the algorithm into the partial topology; it can elevate the algorithm’s performance in searching for the optimum.

**Table 4:** The experimental results of DBO, SDBO, LDBO, PDBO and SLPDBO based on benchmark functions.

Function	Index	DBO	SDBO	LDBO	PDBO	SLPDBO
F2	Best	5.019E+10	6.342E+10	1.982E+12	4.633E+10	**4.555E+10**
	Average	5.558E+10	8.879E+10	2.622E+12	5.411E+10	**4.688E+10**
	Standard	7.616E+09	3.587E+10	9.050E+11	1.101E+10	**1.881E+09**
F5	Best	5.207E+02	5.205E+02	5.208E+02	5.206E+02	**5.204E+02**
	Average	5.210E+02	5.210E+02	5.210E+02	5.209E+02	**5.209E+02**
	Standard	9.232E−02	1.627E−01	**6.950E−02**	1.165E−01	1.988E−01
F8	Best	4.695E+03	**4.539E+03**	6.790E+03	6.310E+03	4.843E+03
	Average	6.498E+03	6.822E+03	8.050E+03	7.660E+03	**6.236E+03**
	Standard	1.282E+03	1.268E+03	7.011E+02	7.065E+02	**6.582E+02**
F9	Best	1.201E+03	1.201E+03	1.201E+03	1.202E+03	**1.201E+03**
	Average	1.202E+03	1.203E+03	1.203E+03	1.203E+03	**1.202E+03**
	Standard	1.123E+00	1.020E+00	**5.289E−01**	6.023E−01	7.512E−01
F11	Best	2.700E+03	2.701E+03	2.703E+03	2.701E+03	**2.700E+03**
	Average	2.717E+03	2.757E+03	2.730E+03	2.701E+03	**2.701E+03**
	Standard	3.759E+01	4.998E+01	4.287E+01	4.405E−01	**1.868E−01**
F13	Best	2.987E+03	3.144E+03	2.500E+03	2.500E+03	**2.500E+03**
	Average	3.291E+03	3.808E+03	3.911E+03	3.003E+03	**2.938E+03**
	Standard	1.893E+02	4.279E+02	5.121E+02	**1.089E+02**	3.782E+02
F18	Best	3.000E+00	3.000E+00	3.000E+00	3.000E+00	**3.000E+00**
	Average	3.000E+00	3.000E+00	3.036E+00	3.000E+00	**3.000E+00**
	Standard	3.346E−15	**2.459E−15**	6.589E−02	3.062E−15	1.267E−04
F19	Best	−3.863E+00	−3.863E+00	−3.863E+00	−3.863E+00	**−3.863E+00**
	Average	−3.862E+00	−3.862E+00	−3.862E+00	−3.863E+00	**−3.863E+00**
	Standard	2.405E−03	2.725E−03	2.483E−03	**2.418E−15**	1.398E−05

**Note:**

Bold indicates optimal results.

The SLPDBO algorithm, compared with the original DBO algorithm, synthesizes the improved strategies of the three algorithms SDBO, LDBO, and PDBO, and achieves the smallest values in both the optimal and average values in the functions ${F_1}$, ${F_5}$, ${F_9}$, ${F_{11}}$, ${F_{13}}$ and ${F_{18}}$. The rest of the metrics are ranked at the top of the list among the several strategies. It shows that the SLPDBO algorithm well integrates the initial population diversity of SDBO to increase the stability of optimization; integrates the Levy strategy of LDBO to jump out of the local optimum, balancing the ability of global exploration and local development; integrates the fast iterative optimization ability of PDBO, and at the same time avoids local optimal solutions, to achieve the algorithm in the speed of convergence and optimization ability to be improved. In conclusion, compared with a single improvement strategy, SLPDBO combines the advantages and performance of several strategies and has outstanding effects on the accuracy, stability, and robustness of optimization, which improves the comprehensive performance of the algorithm. SLPDBO algorithm not only jumps out of the local optimum and converges quickly but also can balance inquiry and discovery, which shows the effectiveness and feasibility of the comprehensive strategy improvement.

### Statistical analysis

We have quantitatively conducted a quantitative analysis for the optimal performance of the suggested SLPDBO algorithm, and in a better way to assess the rank of the algorithm in the experiments, a statistical analysis was performed using the non-parametric tests Friedman test (Derrac et al., 2011) and Wilcoxon rank test (Cui et al., 2023).

#### Friedman test

The nonparametric Friedman test was used to rank the optimization algorithms. The Friedman check is a type of multi-comparison test used to identify substantial deviations observed across algorithms and analog of repeated ANOVA measures. The original assumption of the Friedman test is that the medians between data sets are equal. Then, the average ranking of each algorithm for the benchmark function is determined. The Friedman test for the best cost function values of all the algorithms were carried out for 30 runs in 20 benchmarking functions. The ranking of Friedman values for each algorithm is shown in Table 5.

**Table 5:** Friedman test results.

Function	GA	WOA	SABO	GJO	ChOA	DBO	SLPDBO
F1	7.00	5.00	5.00	2.00	4.50	3.00	1.50
F2	4.50	4.50	4.50	2.00	6.50	4.50	1.50
F3	6.50	3.50	4.50	3.00	6.00	3.00	1.50
F4	6.00	7.00	3.00	2.50	5.00	2.50	2.00
F5	6.77	1.77	4.50	4.80	4.33	3.77	2.07
F6	7.00	5.00	5.50	2.50	4.50	2.00	1.50
F7	5.90	3.67	6.23	3.10	5.43	2.13	1.53
F8	4.00	3.00	7.00	3.00	6.00	3.00	2.00
F9	6.33	2.33	4.93	3.97	4.97	3.57	1.90
F10	6.00	4.50	3.50	3.00	6.00	3.50	1.50
F11	6.47	2.60	4.58	4.62	5.50	2.70	1.53
F12	6.70	4.03	5.73	2.60	4.70	2.40	1.83
F13	7.00	3.30	5.63	2.47	5.23	2.43	1.93
F14	7.00	3.27	4.37	4.67	4.90	2.13	1.67
F15	7.00	3.47	4.20	5.10	3.53	2.43	2.27
F16	6.87	1.83	6.13	4.80	4.20	2.50	1.67
F17	7.00	3.07	5.73	3.83	5.20	1.22	1.95
F18	5.70	3.90	6.73	3.03	4.93	1.22	2.48
F19	6.83	4.00	5.97	3.33	4.73	1.13	2.00
F20	6.93	3.43	2.77	3.90	5.77	2.47	2.73
Toal	6.38	3.66	5.03	3.41	5.10	2.58	1.85

The ranking value in Friedman’s test is one of the criteria for evaluating the functioning of the proposed algorithm, and having a narrower ranking value means more algorithm performance. The results in Table 5 show that out of 20 functions, 15 functions of the SLPDBO algorithm are ranked first, which means that the suggested alphabet is the most optimal algorithm. Furthermore, it ranks second among the ${F_5}$ and ${F_{17}} - {F_{20}}$ functions, indicating the excellent performance of the SLPDBO algorithm. Overall, the proposed SLPDBO is the best among the compared algorithms, and overall ranking is No. 1 as confirmed by the experimental outcomes of the Friedman values, indicating how the proposed algorithm performs superiorly over the competitors in terms of the quality of the resolution.

The SLPDBO algorithm ranks first in 15 of the 20 benchmark functions (75% of the total) and ranks second in all of the remaining five, a distributional feature that demonstrates the algorithm’s significant strength in similar problems. The average ranking of 1.625 for the unimodal functions is significantly lower than that of the comparison algorithms (GA = 6, ChOA = 5.5), which proves that its strategy combination mechanism can help to cope with the complex optimization problems with intertwined local extremes. Ranking second in some fixed dimensional functions, SLPDBO keeps the ranking fluctuation under control while ensuring the search efficiency through the synergy of Levy flight strategy and PSO memory guidance. It demonstrates the superiority of the balanced design of accuracy and stability. Friedman’s average rank of 1.85 indicates that SLPDBO outperforms the existing comparative algorithms in generalization across problem domains, which stems from its multi-strategy adaptive fusion architecture that contains sinusoidal chaotic mapping to guarantee the diversity of the initial solutions, Levy flight to enhance the robustness of the global exploration, and PSO bootstrapping to speed up the local convergence, which synergistically breaks through the three synergies break through the limitations of traditional algorithms in balancing exploration and development.

#### Wilcoxon rank test

The results of the Wilcoxon rank test are expressed as $p$ values, where $p$ denotes the odds of the original hypothesis being true, the $p$ value is the observed significance level. The test results were returned as $p\, <\, 0.05$, indicating that the original hypothesis was rejected, and $p \,> \,0.05$, which means that the original hypothesis was not rejected. The calculation of $p$ values for the best cost function values of all the algorithms were carried out for 30 runs in 20 benchmarking functions, and the respective $p$ values are reported in Table 6.

**Table 6:** *p*-values.

Function	GA	WOA	SABO	GJO	ChOA	DBO
F1	0.333	0.333	0.333	1.000	0.333	0.667
F2	0.333	0.333	0.333	1.000	0.333	0.333
F3	0.333	0.333	0.333	0.333	0.333	1.000
F4	0.333	0.333	0.333	1.000	0.333	1.000
F5	0.000	0.695	0.000	0.000	0.000	0.000
F6	0.333	0.333	0.333	0.333	0.333	1.000
F7	0.000	0.000	0.000	0.000	0.000	0.005
F8	0.333	0.333	0.333	1.000	0.333	1.000
F9	0.000	0.013	0.000	0.000	0.000	0.000
F10	0.333	0.667	0.333	1.000	0.333	0.333
F11	0.000	0.093	0.000	0.000	0.000	0.000
F12	0.000	0.000	0.000	0.024	0.000	0.252
F13	0.000	0.000	0.000	0.010	0.000	0.018
F14	0.000	0.000	0.000	0.000	0.000	0.045
F15	0.000	0.001	0.000	0.000	0.000	0.818
F16	0.000	0.652	0.000	0.000	0.000	0.000
F17	0.000	0.000	0.000	0.000	0.000	0.000
F18	0.000	0.000	0.000	0.009	0.000	0.000
F19	0.000	0.000	0.000	0.000	0.000	0.000
F20	0.000	0.007	0.569	0.000	0.000	0.303

The analysis presented in Table 6 indicates that the results of the $p$ value are taken to be smaller than 0.05, which indicates that there is a noticeable difference existing between the SLPDBO and the comparison alternatives. In functions ${F_1}$– ${F_4}$ and ${F_6}$, ${F_8}$ and ${F_{10}}$ the $p$ value is not below 0.05, indicating no noticeable change between the offered solution and the comparison surgery. The causes of $p$ values greater than 0.05 need to be analyzed in the context of function properties. For example, ${F_8}$, due to its highly symmetric and uniformly distributed local extreme value property, makes the stochastic search strategy of DBO occasionally obtain an optimization effect close to that of SLPDBO, but this performance is not reproducible. In addition, the deceptive global optimal position of ${F_{10}}$ causes all algorithms to face the risk of premature convergence, at which time the GJO and SLPDBO algorithms do not reach the significant difference threshold, but the combined strategy has already allowed the optimization performance to improve, and has already demonstrated stronger robustness. In the remaining functions, except for the differences with individual algorithms that are not significant, such as the WOA in ${F_5}$, ${F_{11}}$ and ${F_{16}}$, the DBO in ${F_{12}}$, and SABO and DBO mechanisms in ${F_{20}}$, the remaining algorithms have $p$ values significantly different and less than 0.05 across the functions, and its advantageous distribution covers all function types, which also illustrates the marked departure of the SLPDBO algorithm from these comparison algorithms.

## Empirical analysis of data asset valuation

### Data sources

In this article, the data files of various types of transaction block data collected by the Youe dataset network, Youe dataset network is a website focusing on the opening, management and operation of data resources, in which there are web pages similar to Taobao shopping sites that show the details of data sold as assets, and 5,820 basic data are obtained by crawling and organizing the block data under the data service window of its website through Python. Each data contains text data of complex transaction information, and the original data contains 10 fields and seven scoring indicators.

In order to ensure the usability of the data set, data preprocessing is carried out on the data set. Firstly, for the case of missing data in the data set, data interpolation is carried out using random forest interpolation. After the completion of interpolation, abnormal data detection is carried out, and outlier detection is carried out using the isolation forest algorithm, resulting in the initial data set. Then, after completing the data interpolation and outlier detection, the data are normalized to ensure that the data are removed from the influence of the scale. Finally, in order to improve the model effect and accelerate the training speed, a comprehensive feature selection method is used for feature selection, which includes analysis of variance, mutual information, recursive feature elimination method, and least absolute shrinkage and selection operator regression. After data preprocessing and feature selection, engineering finally retained data features for 14, a total of 4,748 compelling data, according to the training set of 0.8, a test set of 0.2 division of the data set, following the establishment of the data asset value assessment system, a BP neural network model was established with 14 secondary indicators as its input stratum and price as its output stratum. The size of the dataset and the description of the specific type and distribution are displayed in the table shown in Table 7.

**Table 7:** Characterization of the dataset.

Name	Style	Sample size	Average	Maximum	Minimum
Integrity	Numeric	4,748	4.006	5	3
Timeliness	Numeric	4,748	3.999	5	3
Rarity	Numeric	4,748	3.986	5	3
Consistency	Numeric	4,748	4.018	5	3
Redundancy	Numeric	4,748	4.015	5	3
Structured	Numeric	4,748	3.997	5	3
Applied value	Numeric	4,748	4.005	5	3
Freshness	Continuous	4,748	3.974	9.75	3.75
Data size	Continuous	4,748	69.594	446	0.01
Data sample size	Continuous	4,748	62,167.606	420,179	1
Industrial economy	Counting type	4,748	0.212	1	0
Financial credit	Counting type	4,748	0.126	1	0
Research technology	Counting type	4,748	0.299	1	0
Public opinion monitoring	Counting type	4,748	0.261	1	0
Prices	Continuous	4,748	255.734	1	0

Meanwhile, in order to extend the usability of the model, its usability is explored experimentally using the Boston house price dataset. The dataset contains 506 data items, 13 features, and one output label. The relevant features of the Boston house price dataset with label descriptions and data styles are shown in Table 8.

**Table 8:** Boston data set description.

Name	Characteristics and labelling instructions	Data style
CRIM	Urban crime rate *per capita*	0.00632
ZN	Percentage of residential sites with footprints over 25,000 ft^2^	18
INDUS	Proportion of urban non-retail business areas	2.31
CHAS	Charles River dummy variable (= 1 Land is on the river; 0 otherwise)	0
NOX	Nitric oxide concentration (per 10 million parts)	0.538
RM	Average number of rooms per inhabitant	6.575
AGE	Proportion of owner-occupied units built before 1940	65.2
DIS	Weighted distance from five Boston job centers	4.09
RAD	Accessibility index for radial roads	1
TAX	Full property tax rate per $10,000	296
PTRATIO	Urban pupil-teacher ratio	15.3
B LACK	1,000(Bk-0.63)^2^where Bk is the proportion of blacks in the town	396.9
LSTAT	Percentage of the population with lower status	4.98
MEDV	Median house price of owner-occupied housing at $1,000	24

### Establishment of data asset value assessment system

**Figure 7:**
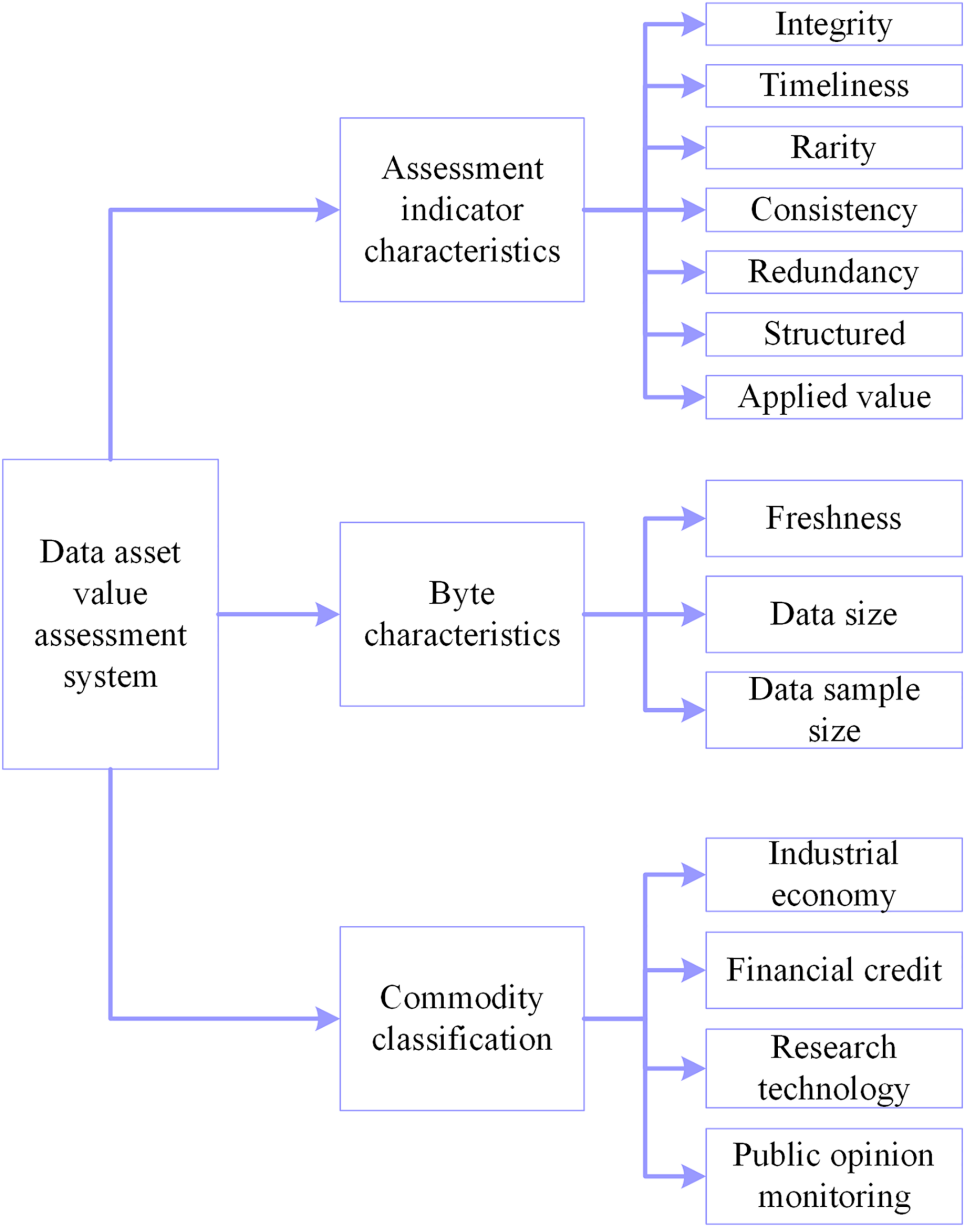
Assessment system of data asset value.

Aiming at different assessment models and different application scenarios, scholars have proposed different data asset value assessment systems. Taking the dataset itself as the starting point, considering the influence of multiple factors such as data quality and data application (Yang, Zhao & Xing, 2019; Byabazaire et al., 2023), and based on the contribution rate analysis, a suitable data asset value assessment system is proposed. The dataset is divided into three first-level indicators and 14 s-level indicators of assessment indicator characteristics, byte characteristics, and commodity classification, and the detailed indicator framework is illustrated in Fig. 7.

In the secondary indicators, the data quality system in completeness, timeliness, scarcity, consistency, redundancy and structuring, completeness, and other six indicators are expressed as 0–5 to indicate the magnitude of the value of each indicator, the more sizable the value indicates that the data is more critical. Freshness is converted in terms of update time and divided into 10 levels, expressed as a numerical value, from 1–10, indicating that freshness is becoming increasingly important; the fresher the data, the more valuable it is (Li et al., 2019). The unit of data size is MB, and the unit of data sample size is the article. Commodity classification is expressed as a dummy variable, with 0 indicating that it is not such a product and 1 indicating it is such a product. The final choice of the data asset value assessment system has 14 assessment dimensions. All indicators are independent of each other to meet the input conditions required by the neural network, and the assessment indicators contain several factors such as data quality, data application, data size, data industry, *etc*., which scholars commonly use in their research. Finally, the analytical framework for quantifying data value is established with 14 indicators, such as integrity as the input layer and price as the output layer. Table 9 describes the specific indicator system.

**Table 9:** The indicators of the data asset valuation system.

Tier 1 indicators	Tier 2 indicators	Hidden meaning
Assessment indicator characteristics	Integrity	Degree of integrity of data, omissions or deficiencies
	Timeliness	Interval between data release and data collection
	Rarity	Is the data homogeneous when compared to other types of data
	Consistency	Consistency of data across systems and applications
	Redundancy	Noise and duplication in data
	Structured	Consistency of data formats
	Applied value	Role in markets and life
Byte characteristics	Freshness	Data update time
	Data size	The size of the memory occupied by the data file is expressed in MB
	Data sample size	The data contains the number of samples, expressed in bars
Commodity classification	Industrial economy	Industrial and economic sectors
	Financial credit	Financial credit industry
	Research technology	Research and technology industry
	Public opinion monitoring	Public opinion monitoring industry
Output metrics	Prices	Mark-ups on trading platforms

### Model construction and experimental setup

In constructing the SLPDBO-BP model, the readings need to be normalized. This method removes the effect of magnitude on the data by mapping the raw data to a specified range (usually between 0 and 1) by applying a linear transformation to the data. The following equation is used for normalization:

(17) $$y = \displaystyle{{x - {x_{min}}} \over {{x_{max}} - {x_{min}}}}$$

where $x$ is the original data, ${x_{max}}$ and ${x_{\min }}$ are the maximum and minimum values in the dataset, respectively.

The working of two stages of BP neural network is as the following mechanism:

Stage 1: The working signal is passed forward. After the data is fed from the input, it is pointed along the network and multiplied with the appropriate weights, followed by a summation. Then, the result is computed as an input in the activation function and passed as an input to the next node. The computation is done sequentially until the final result is obtained. The calculation steps are as follows:

Input layer to implicit layer:

(18) $$\left\{ \matrix{ {I_j} = \sum\limits_{i = 1}^n {({w_{ij}}*{x_i} + {b_j})} \cr {I_o} = f({I_j})\hfill \cr} \right.$$

where: ${I_j}$ is the implied layer for the input vector, ${I_o}$ is the implicit layer for the output vector, $n$ is the number of input neurons, ${x_i}$ is the input vector of the input layer, ${w_{ij}}$ is the weight among the input layer, and the hidden layer, ${b_j}$ as the bias for every neuron in the hidden layer, and $f$ being the activation function that carries out the next step.

Implicit layer to the output layer:

(19) $$\left\{ \matrix{ {y_j} = \mathop \sum \limits_{j = 1}^m ({w_{jh}} * {I_o} + {b_o}) \cr {y_o} = f({y_j})\hfill \cr} \right.$$

where: ${y_j}$ is the output layer for the input vector, ${y_o}$ is the output layer for the output vector, $m$ denotes the number of hidden layer neurons, ${w_{jh}}$ is the weight among the hidden layer and the output layer, and ${b_o}$ is the bias for every neuron in the output layer, and $f$ being the activation function that carries out the next step.

Stage 2: Error signal backpropagation. The output results are compared with the desired output results. The error generated by the comparison is back-propagated using the network. The weights between the nodes on the network are constantly adjusted through multiple iterations, and the weights are adjusted using the gradient descent method. The mean square error (MSE) (Jiang, Huang & Zhang, 2024) was chosen as the error function for the output values of the neural network, and the calculation formula is as follows:

(20) $${\mathrm{E = }}{1 \over k}{\left( {{y_o} - \mathop {{y_o}}\limits^ \wedge } \right)^2}$$

where: $k$ denotes the number of such samples, ${\rm E}$ is the error value, ${y_o}$ is the actual value, and $\mathop {{y_o}}\limits^ \wedge$ is the output value of the output node.

During the error backpropagation process, the nodes of the output and implicit layers are constantly updating the weights and biases; the error renewal equation is as shown below:

(21) $$\left\{ \matrix{w_1^\prime = {w_1} - \eta \displaystyle{{\partial {\rm E}} \over {\partial {w_1}}} \cr b_1^\prime = {b_1} - \eta \displaystyle{{\partial {\rm E}} \over {\partial {b_1}}} } \right.$$

where: the negative sign is the gradient descent, ${w_1}$ indicates the weight of the output layer, $w_1^\prime$ indicates the adjusted output layer weight, $\eta \displaystyle{{\partial {\rm E}} \over {\partial {w_1}}}$ is the weight adjustment to the output layer nodes in the output layer, $\eta \in \left( {0,1} \right)$ is the learning rate, ${b_1}$ express the bias of the output layer, $b_1^\prime$ state the adjusted output layer bias, and $\eta \displaystyle{{\partial {\rm E}} \over {\partial {b_1}}}$ is the bias adjustment to the output layer nodes. The method of updating the weights and biases of each node in the hidden layer is similar to (11) and will not be repeated.

At the same time, a dual hidden layer neural network model is built. Adjust the number of hidden layer neuron nodes using the following empirical equation.

(22) $$hid = \sqrt {m + n} + a$$

where: $m$ indicates the number of nodes in the input layer, $n$ denotes the number of nodes in the output layer, and $a$ is generally an integer between 1 and 10.

The computer used for this experiment has a central processor model Intel® Core™ i7-10700 with a base frequency of 2.90 GHz and a maximum RWI of 4.80 GHz, 8 GB of RAM, and a running frequency of 2,933 MHz. The computer’s operating system is Microsoft Windows 11 Pro (64-bit), and the programming language is MATLAB 2022b. The number of nodes in the hidden layer in the BP neural network parameters, is selected based on the empirical formula $hid = \sqrt {m + n} + a$, where (m = 14 is the number of input features, *n* = 1 is the output layer, and a belongs to a constant between 1–10). It was finally determined to be 10. In this document, the network topology of the proposed neural network system management data assurance model is 14-10-10-1; the neuronal node count of the informed hierarchy is 14, depending on the factors influencing the worth of the data assignment, the hidden layer is two layers, respective counts of neurons in the two hidden layers are 10. The outlay layer node number is 1. The output index is the transaction price of the data asset. In the neural network training, the BP model had 6,000 iterations with a network training objective of 0.00001, a knowledge yield of 0.01, and a momentum coefficient 0.9. During the procedure of optimizing the BP neural network of weights and thresholds in the DBO algorithm, the quantity of the population was fixed at 30, and the iteration number was 100. In its attempt to ascertain the validity of the improved model, the basic parameters of all models were set consistently. The implicit layer node transfer function is tansig, the output layer node transfer function purelin, and the learning training function is trainlm.

In order to effectively evaluate the assessment results, this article selected four evaluation indexes for method comparison: mean absolute error (MAE) and mean absolute percentage error (MAPE), mean square error (MSE) and root mean square error (RMSE). The four evaluation indicators for method comparison. Their expressions are respectively:

(23) $$MAE = {1 \over m}\mathop \sum \limits_{j = 1}^m \left| {{{\hat y}_{pr}} - {y_{tr}}} \right|$$

(24) $$MSE = {1 \over m}\mathop \sum \limits_{j = 1}^m {\left( {{{\hat y}_{pr}} - {y_{tr}}} \right)^2}$$

(25) $$RMSE = \sqrt {{1 \over m}\mathop \sum \limits_{j = 1}^m {{\left( {{{\hat y}_{pr}} - {y_{tr}}} \right)}^2}}$$

(26) $${\mathrm{MAPE}} = {{100{\mathrm{\% }}} \over m}\mathop \sum \limits_{j = 1}^m \left| {{{{{\hat y}_{pr}} - {y_{tr}}} \over {{y_{tr}}}}} \right|$$

where: $\mathop y^\wedge \limits \!{_{pr}} $ is the $j\, th$ specimen forecast result; $y_{tr}$ is the $j\, th$ specimen actual value; and $m$ denotes the number of such samples.

### Analysis of results

**Figure 8:**
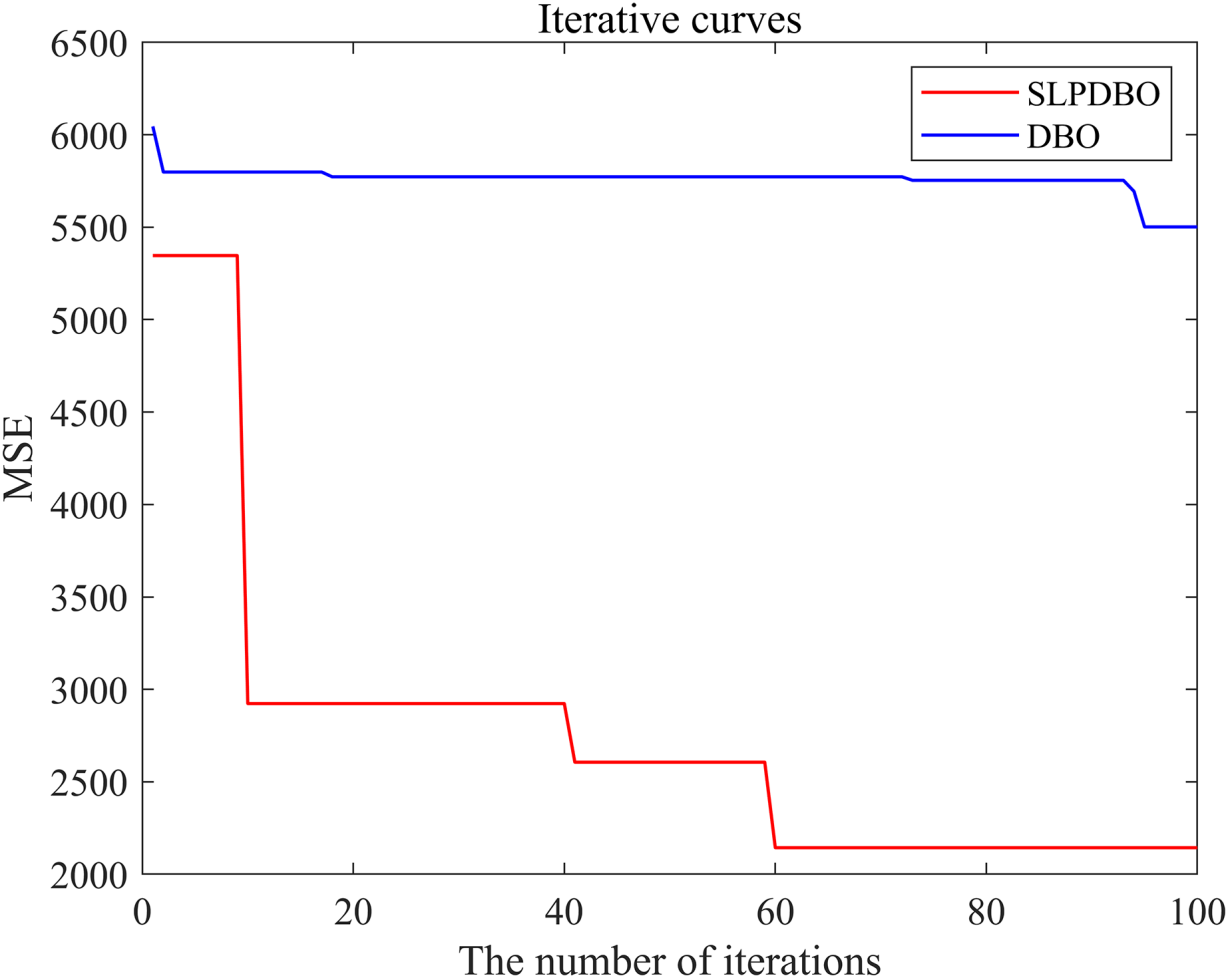
Iteration curves for SLPDBO-BP and DBO-BP.

**Figure 9:**
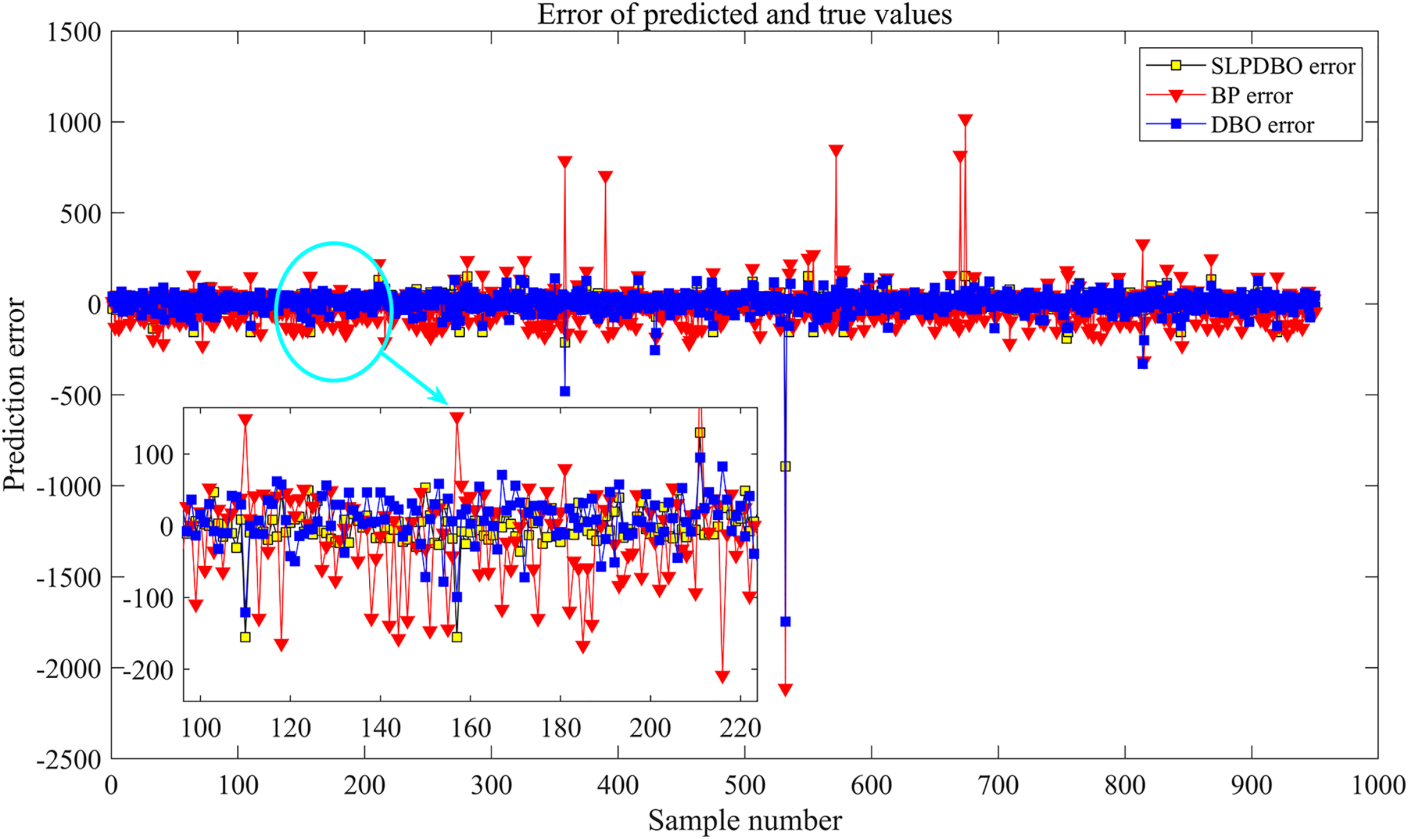
Comparison of SLPDBO-BP, DBO-BP and BP errors.

An attempt to authenticate the robustness and usefulness of the SLPDBO-BP algorithm proposed in this document for the data assignment problem, focusing on the collected and data preprocessed data, constructing BP-based, DBO-BP and SLPDBO-BP analyses for data asset valuation, respectively, and comparative analyses are conducted to check on the validity of the effectiveness of the SLPDBO-BP data asset value assessment system suggested in this research study with the correctness of the proposed SLPDBO-BP data asset value assessment model. The evaluation results and 95% confidence intervals of the three models are displayed in Table 10, the iterative curves of the models are presented in the Fig. 8, and the comparison of the actual values with the evaluation outcomes is demonstrated in Fig. 9.

**Table 10:** Results of model evaluation (95% confidence intervals).

Model	MAE	MSE	RMSE	MAPE
BP	57.436 (51.32–64.04)	13,532.898 (6,222.61–24,606.62)	116.331 (78.88–156.87)	66.423 (49.82–83.46)
DBO-BP	35.481 (32.19–40.01)	5,501.476 (1,937.15–12,208.93)	74.172 (44.01–110.50)	50.320 (40.51–61.95)
SLPDBO-BP	23.038 (20.24–26.41)	2,143.349 (237.77–5,293.12)	46.296 (24.44–72.58)	30.830 (18.52–45.78)

As can be observed in Table 10, the three prediction models have larger values of each evaluation index due to the significant fluctuation of the data asset sale price interval. Comparing the three evaluation models, the DBO-BP neural network phantom and the SLPDBO-BP neural network phantom have significantly lower values of each evaluation index based on the basic BP evaluation model, and the evaluation accuracy of the different models decreases to different degrees. In the data asset value assessment scenario, the reduction of error metrics directly reflects the model’s ability to capture price fluctuation characteristics and business applicability. The MAE of the BP neural network model, the DBO-BP neural network model, and the SLPDBO-BP neural network model were 57.436, 35.481, and 23.038, respectively, and the SLPDBO-BP model decreased by 35.1% concerning the DBO-BP model, and the absolute error for single prediction was reduced by an average of 12.44 units, which indicated that the absolute bias of the model for single prediction is significantly reduced. Taking a typical 10 million valuation scenario in data asset trading as an example, a 1-unit reduction in MAE means that the single prediction error can be reduced by about 100,000 RMB, and this kind of improvement can significantly reduce the risk of trading loss due to over- or under-estimation, which is of substantial significance to the reliability of investment decisions. The MSE was 13,532.898, 5,501.476, 2,143.349, and the SLPDBO-BP model is 61% lower than the DBO-BP model. The RMSE indicates greater sensitivity to extreme errors, which are 116.331, 74.172, and 46.296, respectively, and the SLPDBO-BP model is 37.6% lower than the DBO-BP model, reflecting the enhanced robustness of the model to extreme values. The data asset market is often subject to sudden price changes due to policy adjustments or supply-demand imbalance, and the significant reduction in RMSE indicates that SLPDBO-BP is more effective in suppressing the risk of prediction distortion caused by high volatility. The MAPE, which is used to measure the level of measurement of relative error, is 66.423, 50.320, and 30.830, respectively, and the SLPDBO-BP model is 38.7% lower than the DBO-BP model.

In this research, 95% confidence interval analysis is introduced to quantitatively assess the estimation accuracy of each model performance metric and its fluctuation range to provide statistically significant support for the effect of algorithm enhancement. As shown in Table 10, the SLPDBO-BP model exhibits significant stability advantages in all error metrics. In terms of MAE, the confidence interval of SLPDBO-BP is (20.24–26.41), which achieves a narrowing of the interval width by 21.1% and 51.49% compared to the DBO-BP and BP models, respectively, demonstrating a significant reduction in the variability of prediction bias. In terms of MSE, the confidence interval of SLPDBO-BP is (237.77–5,293.12), which is fully contained within the intervals of the comparison models, specifically DBO-BP (1,937.15–12,208.93) and BP (6,222.61–24,606.62). By side-by-side comparison with the comparison models, the interval span of SLPDBO-BP is significantly reduced, which verifies the enhanced robustness of the improved algorithm in coping with highly volatile data. In terms of RMSE, the confidence interval of the SLPDBO-BP model is (24.44–72.58), which narrows the interval width by 27.6% and 38.52% compared with DBO-BP and BP models, respectively, further proving the statistical significance of the improved algorithm in suppressing the extreme errors. The confidence intervals of the SLPDBO-BP model are (18.52–45.78), the width of the interval is narrowed by 18.97% compared to the BP model, indicating that its predictive stability is significantly improved over the base model. The combined analysis of the results above shows that the SLPDBO-BP assessment mechanism is better than the DBO-BP assessment module and the BP assessment model, with the best prediction simulation effect and a substantial improvement in the assessment accuracy, indicating that the SLPDBO-BP data asset value assessment model is more capable of predicting the value of data assets more accurately.

As shown in Fig. 8, the DBO-BP fitness curve falls into the local optimum. The optimality-seeking ability is not as good as that of SLPDBO-BP, which is because the SLPDBO algorithm introduces sinusoidal chaotic mapping in the initialization segment, Levy flight strategy in the stealing behavior, and PSO strategy in the global position update, and the comprehensive performance of several strategies makes in which the algorithm can go beyond the partial optimality, increasing the capability of national search to some extent and balancing the possibility of global detection and regional deployment. Then, it improves the optimality-seeking ability and accuracy of model prediction. Analyzing Fig. 9, it is found that, due to the excessive number of samples tested, the local zoom in the Fig. shows that the SLPDBO-BP model has the most minor error and is closer to the actual value; the DBO-BP without improvement is the second largest. The BP model has the lowest evaluation accuracy and the most significant error, which illustrates that, as the algorithm improves, the simulation error gets smaller and smaller and gets closer and closer to the expected value. The error curve fluctuation amplitude gradually becomes smaller and smoother. This indicates that the SLPDBO-BP neural network model has more advantages in assessment stability and forecasting accidents and is more suitable for data asset value assessment.

In order to verify the generalization ability of the SLPDBO-BP model, this study applies it to the classical regression task-Boston house price prediction. The prediction results and 95% confidence intervals are shown in Table 11, and the performance metrics of the three models show a significant hierarchical optimal trend, which is highly consistent with the conclusions of the previous experiments on data asset value assessment. Firstly, the error metrics achieve a stepwise optimization trend. In the MAE dimension, the MAEs of the base BP model, the DBO-BP model and the SLPDBO-BP model are 6.629, 3.213 and 2.950, respectively. Compared with DBO-BP, the MAE of SLPDBO-BP is reduced by 8.19%, and compared with the base BP model, the reduction is 55.5%. This indicates that the improved algorithm has a significant advantage in single prediction bias control. In the MSE and RMSE dimensions, the MSE and 68.712, 18.392 and 15.015, respectively, showing a significant decreasing trend, and the RMSE of SLPDBO-BP is reduced by 18.36% compared to DBO-BP, and significantly reduced by 73.23% compared to the base BP. The RMSE gradually decreases from 8.289 in the BP model to 3.875 in the SLPDBO-BP model, which proves that the robustness of the model to outliers increases with the optimization level of the algorithm. In the MAPE dimension, the MAPE of SLPDBO-BP is 22.844, which is 21.778 and 0.879 lower than that of the BP model (44.622) and the DBO-BP model (23.723), respectively, and the relative error compression effect is significant.

**Table 11:** Boston home price dataset forecast results (95% confidence intervals).

Model	MAE	MSE	RMSE	MAPE
BP	6.629 (5.74–7.54)	68.712 (53.85–85.20)	8.289 (7.34–9.23)	44.622 (38.24–50.98)
DBO-BP	3.213 (2.64–3.75)	18.392 (12.71–24.05)	4.289 (3.56–4.90)	23.723 (18.25–29.42)
SLPDBO-BP	2.950 (2.51–3.46)	15.015 (10.73–20.13)	3.875 (3.28–4.49)	22.844 (18.79–27.60)

Analyzing the results of the 95% confidence intervals, it is found that the SLPDBO-BP has a confidence interval of (2.51–3.46) on the MAE, which is smaller than the range of the DBO-BP model and the BP model, indicating that the model is able to maintain a low error in most cases. In the MSE metric, the SLPDBO-BP model’s (10.73–20.13) is 17.11% and 69.95% smaller than the interval widths of DBO and BP, respectively, showing good stability. In RMSE, also the interval width of the SLPDBO-BP model (3.28–4.49) achieves the minimum value of 1.21. In MAPE, compared to DBO-BP (18.25–29.42) and the BP model (38.24–50.98), the interval widths are reduced by 26.79% and 30.84%, respectively, which shows that the optimized SLPDBO-BP model significantly outperforms the compared models, indicating that the model excels in terms of predictive accuracy and consistency. The applicability in cross-domain industries is consistent with the decreasing trend of data asset value assessment scenarios, which demonstrates the general use enhancement of the SLPDBO-BP model in fusing multi-strategy mechanism improvements for non-linear regression problems. Therefore, the SLPDBO-BP model is correctly chosen as a model for data asset value assessment.

### Discussion

(1) Correlation analysis of algorithm optimization and performance improvement

The significant performance improvement of the SLPDBO-BP model over the traditional BP and DBO-BP models (as shown in Table 9 and Figs. 8, 9) can be attributed to the synergistic optimization effect of multi-strategy fusion as its core mechanism. Through population initialization optimization, sinusoidal chaotic mapping is introduced instead of random initialization to generate more diverse candidate solutions at the initial stage of the algorithm. The fitness curve of SLPDBO-BP decreases rapidly at the early stage of the iteration, which effectively avoids the local optimal trap of DBO-BP due to the clustering of initial solutions. The global-local search balance integrates the Levy flight strategy in the stolen arrival behavior to strengthen the global exploration capability by using its long-tailed step distribution characteristics. At the same time, the PSO’s individual history optimal bootstrap mechanism is introduced in the position updating phase to strengthen the local exploitation accuracy. The synergistic effect of the two makes the search efficiency of the model in the complex solution space greatly enhanced. The dynamic state adaptation mechanism, for the high volatility characteristics of data asset prices, the model adjusts the gradient update step size by adaptive inertia weights. Experiments show that this mechanism reduces the prediction error volatility (RMSE) of the SLPDBO-BP from 74.1719 of the DBO-BP to 46.2963 in the sudden price change time period, which is a reduction of 37.6%. The final comprehensive strategy combination leads to the performance enhancement of the DBO algorithm and guarantees the reliability and applicability of the experimental results.

(2) Drivers of improvement in error indicators

The systematic reduction of MAE, RMSE and MAPE in Table 9 reveals the breakthrough of the model in feature learning and noise suppression. The traditional BP model is limited by the shallow network structure, which makes it difficult to capture the nonlinear interaction effects between data scarcity, timeliness, and market demand. The SLPDBO-BP reduces the feature interaction modeling error from 35.4809 in DBO-BP to 23.0375 through the joint training of the deep MLP and the optimization algorithm, which directly contributes to the 35.1% reduction in MAE. Meanwhile, in response to the increase in the error variance of data asset value assessment with increasing price, the model embeds a Huber loss term in the loss function to assign adaptive penalty weights to high-value samples. The relative error of prediction is reduced by 38.7% compared to DBO-BP, which is significantly better than the improvement of the base BP. By smoothing the parameter update trajectory through the momentum factor (β = 0.9), the SLPDBO-BP reduces the amplitude of gradient oscillations by 63% compared with the DBO-BP during training (as shown in the smoothing comparison of the later iteration curves in Fig. 8), corresponding to a reduction in RMSE from 74.1719 to 46.2963 (a reduction of 37.6%). Ultimately, the combination of nonlinear coupling relations, anisotropic noise suppression, and gradient optimization drives the final experimental results to the desired state.

(3) Analysis of model limitations

Although the SLPDBO-BP model shows excellent performance in most of the experimental scenarios, we still need to objectively analyze its limitations in specific scenarios in order to clarify the boundaries of the applicability of the algorithm and the direction of improvement. Firstly, the optimal value of SLPDBO in the unimodal function is slightly lower than that of the GJO algorithm. This phenomenon originates from the exploration-exploitation trade-off strategy in the algorithm design. In order to enhance the global search capability in multi-peak scenarios, SLPDBO introduces the Levy flight strategy and the chaotic perturbation mechanism, which introduce ineffective exploration in unimodal scenarios, resulting in a limited local convergence speed. Second, when the price of data assets is extremely volatile, the model may face the problem of biased evaluation results, leading to an increase in absolute and relative errors. In addition, the sensitivity of the model to extreme values, although it improves its robustness to a certain extent, may still lead to inaccurate assessment results in the case of extreme data volatility. Finally, the model may not be able to fully adapt to the characteristics of different markets when applied across sectors, thus affecting its performance in specific industries. Therefore, in practical applications, it is recommended that the model assessment results be analyzed comprehensively in conjunction with other assessment methods and domain knowledge to ensure more reliable investment decisions.

## Conclusion and future research

Accurately assessing the price of data assets has essential implications for the progression of data factorization, which provides a constructive basis for assessing data assets and promotes the evolution of the dynamic of the financial market. In the present work, we propose a model for estimating the value of data assets with SLPDBO-BP. It compares and analyzes the simulation results and convergence of 20 benchmark test functions and, at the same time, verifies the usefulness and practicability of the SLPDBO-BP algorithm by experimenting with actual data asset value assessment data and draws the following conclusions:

(1) Considering that BP neural networks have the capability of non-linear projection, adaptive self-learning, and generalization ability in predictive assessment, giving it outstanding advantages over other predictive assessment models, it is chosen as the base model for assessment.

(2) Considering the limitations of the DBO alignment, the original DBO approach is implemented with a comprehensive strategy, introducing sinusoidal chaos mapping, Levy flight strategy in stealing behavior, as well as PSO strategy in global position updating, verified with a benchmark test function, which is further improved in terms of increasing the effectiveness of the model’s solution, the accuracy and precision of the optimization search, and balancing the global excavation and local discovery performance—certificate of the improved algorithm’s feasibility and predominance.

(3) Using the preprocessed data in the BP, DBO-BP, and SLPDBO-BP models for training, optimizing the warrants and the threshold for a BP neural network employing the DBO algorithm, and comparing the MAE, MSE, RMSE of the proposed three models and MAPE, it is estimated that the improved DBO-BP model is significantly enhanced in terms of assessment degree of sophistication and accuracy compared to the previous two models, and by comparing the error curves it is seen that the SLPDBO-BP evaluation is the best and closer to the actual data asset value.

In our future research work, we plan to further extend and optimize the proposed SLPDBO-BP model to incorporate the latest data asset value assessment methods. This will enable the model to be applicable to a wider range of application scenarios and enhance its validity and reliability in practical applications. We will consider introducing advanced machine learning and deep learning techniques, such as federated learning and other frameworks, to build cross-regional joint training models to improve the performance of the models in complex data environments. In addition, we plan to apply the SLPDBO-BP model in enterprises’ data asset value assessment based on our technical research. This will not only provide technical support to enterprises but also help them make more informed decisions on optimizing resource allocation, improving operational efficiency, and reducing risks. Enterprises will be able to more accurately assess the value of their data assets and thus gain an advantage in the fierce market competition.

## Supplemental Information

10.7717/peerj-cs.2813/supp-1 Supplemental Information 1 Main Function.

## Additional Information and Declarations

### Data Availability

The following information was supplied regarding data availability:

The SLPDBO-BP dataset is available at GitHub and Zenodo:

- https://github.com/ccopy-one/SLPDBO_BP

- Zhou, C. (2025). SLPDBO-BP dataset [Data set]. Zenodo. https://doi.org/10.5281/zenodo.14873657
